# AZD8701, an Antisense Oligonucleotide Targeting FOXP3 mRNA, as Monotherapy and in Combination with Durvalumab: A Phase I Trial in Patients with Advanced Solid Tumors

**DOI:** 10.1158/1078-0432.CCR-24-1818

**Published:** 2025-02-12

**Authors:** Lillian L. Siu, Sophie Postel-Vinay, Rafael Villanueva-Vázquez, Guillermo de Velasco, Eduardo Castanon Alvarez, Christos E. Kyriakopoulos, Melissa Johnson, Kaïssa Ouali, Stephen McMorn, Helen K. Angell, Felicia Ng, Shashank Saran, Mahdiye Bayat, Teresa Collins, Archana Roy, Arthur W. Lambert, Song Cho, Neil Miller, Michele Petruzzelli, John Stone, Christophe Massard

**Affiliations:** 1 Princess Margaret Cancer Centre, Toronto, Canada.; 2 Institut Gustave Roussy, Villejuif, France.; 3 Institut Català d'Oncologia, Early Drug Development Unit, Medical Oncology Department, ICO-Hospitalet, Barcelona, Spain.; 4 Hospital Universitario 12 de Octubre, Medical Oncology, Madrid, Spain.; 5 Clínica Universidad de Navarra, Medical Oncology, Madrid, Spain.; 6 Department of Medicine, University of Wisconsin, Madison, Wisconsin.; 7 Lung Cancer Research, Sarah Cannon Research Institute at Tennessee Oncology, Nashville, Tennessee.; 8 Drug Development Department, Institut Gustave Roussy, Villejuif, France.; 9 AstraZeneca, Cambridge, United Kingdom.; 10 AstraZeneca, Gaithersburg, Maryland.; 11 AstraZeneca, Waltham, Massachusetts.; 12 DITEP, Institut Gustave Roussy, Villejuif, France.; 13 Faculty of Medicine, Paris Saclay University, Paris, France.; 14 Molecular Radiotherapy Unit 1030, National Institute of Health and Medical Research (INSERM), Paris, France.

## Abstract

**Purpose:**

AZD8701 uses next-generation antisense oligonucleotide (ASO) technology to selectively reduce human forkhead box P3 (FOXP3) expression in regulatory T cells, reversing their immunosuppressive function. *FOXP3* ASO alone or with PD-(L)1 inhibition attenuated tumor growth in mice. We report a phase I study of AZD8701 alone or combined with durvalumab in patients with advanced solid tumors.

**Patients and Methods:**

Eligible patients had solid tumors and received prior standard-of-care treatment, including anti–PD-(L)1 therapy. Patient cohorts were treated with AZD8701 intravenously weekly at escalating doses, either alone (60–960 mg) or combined (240–720 mg) with durvalumab 1,500 mg intravenous every 4 weeks. The primary objective was safety and tolerability, with the aim of determining the MTD.

**Results:**

Forty-five patients received AZD8701 monotherapy, and 18 received AZD8701 with durvalumab. One dose-limiting toxicity (increased alanine aminotransferase) occurred with AZD8701 960 mg. The most common adverse events related to AZD8701 monotherapy were fatigue (22.2%), asthenia, pyrexia, and increased alanine aminotransferase (20% each); the safety profile was similar when combined with durvalumab. With AZD8701 monotherapy, 24.4% and 15.6% of the patients had stable disease for ≥16 and ≥24 weeks, respectively; one patient treated with AZD8701 720 mg and durvalumab had a partial response. *FOXP3* mRNA changes were heterogeneous (8/13 patients showed a reduction), with no clear dose relationship. ASO accumulated in the tumor epithelium and stroma.

**Conclusions:**

This study demonstrates the clinical feasibility of ASO therapy, with generally manageable adverse events, *FOXP3* knockdown, and ASO delivery to the tumor.

**Translational Relevance**

New immunotherapeutic approaches are needed to improve outcomes in patients who do not benefit long-term from PD-(L)1 inhibition. AZD8701 is a novel phosphorothioate-modified chimeric antisense oligonucleotide designed to downregulate human *forkhead box P3 (FOXP3)* mRNA and subsequently reduce target protein levels associated with the immunosuppressive properties of regulatory T cells. In this first-in-human study, we investigated AZD8701 as a potential anticancer therapy for patients with advanced solid tumors. We demonstrated that AZD8701 dose escalation up to 720 mg as monotherapy and in combination with durvalumab was well tolerated by patients with advanced solid tumors. An analysis of *FOXP3* mRNA expression in tumor samples taken pre- and posttreatment showed a modest reduction in *FOXP3* expression that was heterogeneous across patients treated with AZD8701. These findings demonstrate the feasibility of using antisense oligonucleotide therapy clinically and support the proposed mechanism by which AZD8701 targets *FOXP3*.

## Introduction

Immunotherapies, such as PD-(L)1 inhibition, have improved clinical outcomes for patients with advanced cancer. However, a significant proportion of patients with even the most responsive tumor types [non–small cell lung cancer (NSCLC), melanoma, and renal cell carcinoma (RCC)] who initially respond often relapse because of acquired resistance (1–5). New approaches that increase the likelihood of benefit for patients with primary and acquired resistance to available immunotherapies are needed, including novel agents targeting immunosuppressive regulatory T cells (Treg; ref. 4) and synergistic combinations of therapies targeting immunosuppressive Tregs and other parts of the immune system, including PD-(L)1.

Tregs are an integral component of the adaptive immune system, maintaining tolerance to self-antigens and preventing autoimmune diseases. However, they may also contribute to cancer progression by suppressing the body’s antitumor immunity (6, 7). Strategies to control Treg-mediated immunosuppression by targeting molecules expressed by Tregs, such as cytotoxic T-lymphocyte–associated antigen 4, have been tested (8). However, none of these approaches were designed to specifically reverse Treg-mediated immunosuppression and few control all Tregs.

Forkhead box P3 (FOXP3), a member of the forkhead box family of transcription factors, is essential for the lineage specification of Tregs and is the key driver of the gene expression program underlying the immune suppressive function of Tregs (9–11). Immunosuppressive FOXP3-positive Tregs are present in the tumor microenvironment (TME) in numerous solid and hematologic malignancies (7) and have been linked to poor prognosis (12) and a lack of response to PD-(L)1 checkpoint inhibition (13, 14). Targeting FOXP3 may allow for a productive antitumor immune response by selectively impairing the immunosuppressive function of Tregs in the TME.

To date, targeting transcription factors using conventional drug modalities has been a challenge. AZD8701 employs next-generation antisense oligonucleotide (ASO) technology [Gen 2.5 cEtmodified-ASO (15, 16), developed by Ionis Pharmaceuticals] to selectively reduce human *FOXP3* mRNA expression levels. ASO bind to cognate mRNA with high affinity and selectivity, and the targeted mRNA in the resulting duplex is cleaved by RNase H, leading to reductions in mRNA and target protein expression. Preclinical studies have demonstrated the potential of *FOXP3* ASO such as AZD8701 to target tumor-infiltrating Tregs (17). Mouse *FOXP3*-specific ASO produced a significant dose-dependent reduction in *FOXP3* mRNA and FOXP3 protein *in vitro* and *in vivo*. FOXP3 knockdown resulted in the loss of immunosuppressive markers and reduced the immunosuppressive capacity of Tregs in primary cell assays. Tumor growth was significantly attenuated in syngeneic tumor-bearing mice treated with mouse *FOXP3* ASO, with a partial reduction in *FOXP3* mRNA (20%–60% compared with baseline). Moreover, tumor growth inhibition was enhanced when *FOXP3* ASO were combined with PD-(L)1 checkpoint inhibition, again with only partial *FOXP3* mRNA reduction. A partial reduction in *FOXP3* levels is assumed to be beneficial to patient safety, especially when considering the link between loss-of-function mutations in *FOXP3* and autoimmune disorders in humans, the most extreme of which is the very rare immunodysregulation polyendocrinopathy enteropathy X-linked syndrome (18). Significant tumor growth attenuation could be achieved with only partial reduction in *FOXP3* mRNA in preclinical studies (17), indicating that AZD8701 may provide clinical antitumor activity at levels of FOXP3 reduction that do not impact self-tolerance. Preclinical toxicology studies supported this hypothesis: AZD8701 monotherapy at doses up to 30 mg/kg (intravenous or subcutaneous) decreased *FOXP3* mRNA levels in the liver of cynomolgus monkeys and in the spleen of mice with no apparent pharmacologic or toxicologic effects (AstraZeneca data on file).

We report a first-in-human phase I study designed to define the MTD, optimal biological dose (OBD), and/or maximum feasible dose (MFD) of AZD8701, alone or in combination with durvalumab, to inform decision-making regarding monotherapy and anti–PD-(L)1 agent combinations in later-phase clinical studies.

## Patients and Methods

### Study design and treatment

**Figure 1.**
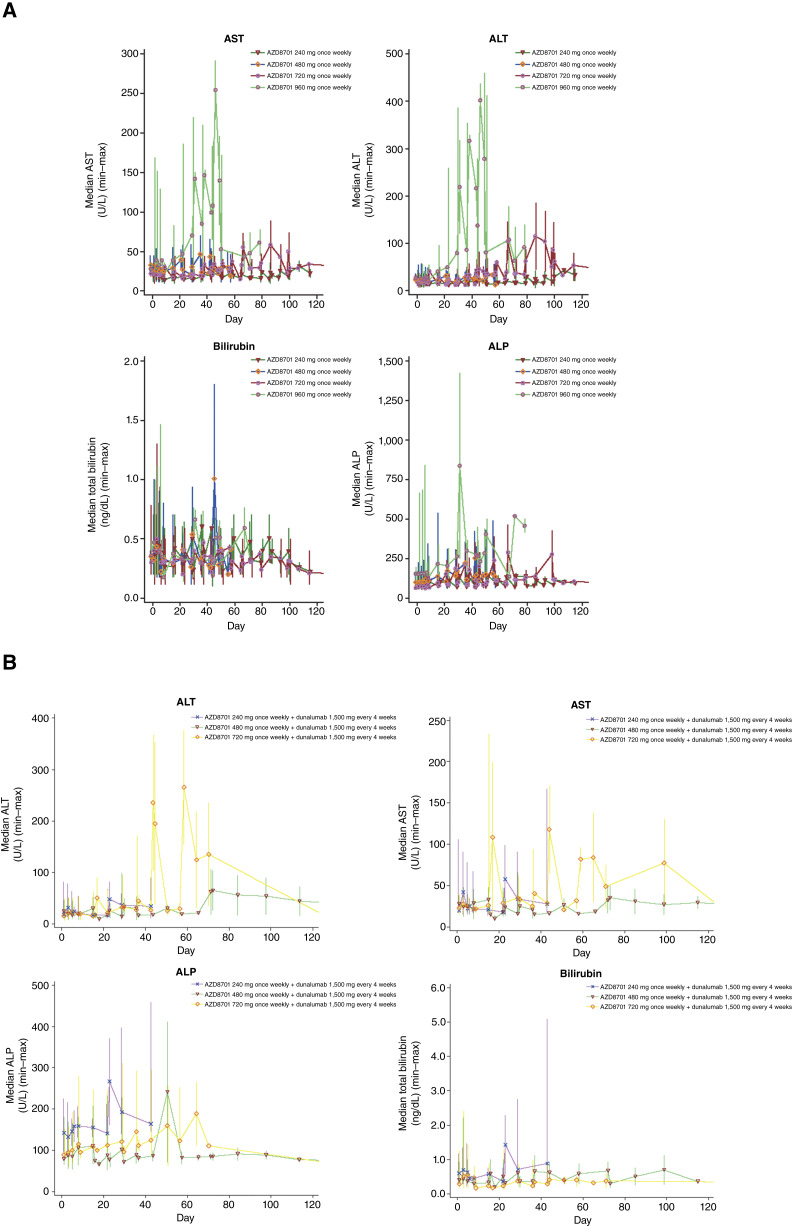
Changes in liver function parameters over time in patients treated with (**A**) AZD8701 monotherapy and (**B**) AZD8701 and durvalumab. ALP, alkaline phosphatase.

This was a phase I, multicenter, open-label, dose-escalation (parts 1 and 3; Fig. 1A), and disease-expansion (parts 2 and 4; Supplementary Fig. S1A) study of AZD8701 alone (parts 1 and 2) or in combination with durvalumab (parts 3 and 4) in patients with selected advanced solid tumors (NCT04504669). The dose-escalation parts (parts 1 and 3) of the study were completed; the planned disease-expansion parts (parts 2 and 4) were not initiated.

AZD8701 was administered by intravenous infusion, with loading doses administered on days 1, 3, and 5 and then once weekly from day 8 of the first 28-day cycle. From cycle 2 onward, AZD8701 was administered once weekly. This dosing schedule was based on data from preclinical studies showing that the use of loading doses achieved rapid steady-state tissue distributions and sustained FOXP3 knockdown for up to 12 weeks and on modeling to predict a clinically active dose range. The starting dose of 60 mg AZD8701 for the monotherapy dose-escalation arm (part 1A) was selected based on the no-observed-adverse-effect level in monkeys. An accelerated titration design based on clinical data for other ASO allowed AZD8701 dose escalation with single-patient cohorts up to and including 120 mg once weekly, provided that (i) this was recommended by the Dose Escalation Committee; (ii) no NCI Common Terminology Criteria for Adverse Events (CTCAE) drug-related toxicity of grade ≥2 was observed; and (iii) no adverse events (AE) that met the dose-limiting toxicity (DLT) criteria (see Supplementary Material S1) was observed during the DLT evaluation period. At dose levels of AZD8701 240 mg once weekly and above, each cohort had to enroll three to six DLT-evaluable patients, with dose escalation following a Bayesian adaptive design scheme (19) until the MTD, and/or OBD, or until MFD was reached.

Once dose escalation reached a level deemed by the Dose Escalation Committee to be tolerable and in a biologically effective range based on study or modeling data [including efficacy, pharmacodynamics (PD), and pharmacokinetics (PK)], three to six additional patients provided paired biopsies to determine dose response (part 1B). In addition, AZD8701 monotherapy was evaluated in approximately eight patients with selected advanced solid tumors [squamous cell carcinoma of the head and neck, triple-negative breast cancer, clear-cell RCC (ccRCC), and NSCLC], who provided mandatory paired biopsies to assess *FOXP3* mRNA modulation (part 1C). This analysis could also include patients from the dose-escalation cohort.

In the combination treatment dose-escalation cohorts (part 3A), each AZD8701 dose cohort consisted of three to six DLT-evaluable patients, with escalation according to a Bayesian adaptive design scheme (19). The standard durvalumab dose of 1,500 mg every 4 weeks intravenously was administered 1 hour after AZD8701 intravenous infusion was completed when these agents were administered on the same day. The AZD8701 starting dose in combination with durvalumab was two levels below the tolerable AZD8701 monotherapy dose determined in part 1A, and no AZD8701 dose escalation above the MTD/OBD/MFD for AZD8701 monotherapy was allowed.

Patients in all parts of the study were treated for a maximum of 2 years or until disease progression, withdrawal of consent, unacceptable toxicity, or other reasons for treatment discontinuation.

The study was conducted in accordance with consensus ethical principles derived from the Declaration of Helsinki, Council for International Organizations of Medical Sciences International Ethical Guidelines, applicable International Council for Harmonization of Technical Requirements for Pharmaceuticals for Human Use (ICH) good clinical practice guidelines, and applicable laws. The protocol was approved by the independent ethics committees and institutional review boards at each study site. All patients provided written informed consent.

### Study population

Eligible patients were ≥18 years old and had histologically or cytologically confirmed solid tumors, including squamous cell carcinoma of the head and neck, triple-negative breast cancer, NSCLC, ccRCC, gastroesophageal cancer, melanoma, cervical cancer, or small cell lung cancer. These tumor types were selected as potentially being the most informative for the development of AZD8701, as they are known to have high Treg infiltration and to be clinically responsive to checkpoint inhibitors (20–24). To broaden inclusiveness, patients with other solid tumors were eligible if they had received >18 weeks of prior anti–PD(L)-1 treatment, based on the Society for Immunotherapy of Cancer consensus clinical definition of acquired resistance to immunotherapy (25). Patients had to have progressive disease refractory to standard therapies, or for which no standard therapies existed at the time of the study. All patients had to have an Eastern Cooperative Oncology Group performance status <2, a life expectancy of ≥12 weeks, at least one qualifying RECIST version 1.1 target lesion, and adequate organ system function. A complete list of inclusion and exclusion criteria is provided in the Supplementary Material S1.

### Study objectives

The primary objectives were to assess the safety and tolerability, characterize DLT, and determine the OBD, MTD, and/or MFD of AZD8701 monotherapy or in combination with durvalumab. The latter objective (determination of OBD, MTD, and/or MFD) was based on a pragmatic approach, given the lack of data on the measurement of *FOXP3* engagement in tumor tissue, which had not been tested clinically. OBD, defined as the lowest safe dose with modeled or measured optimal biologic effect, was the preferred measure, but trying to establish the MTD was judged to be appropriate to achieve greater FOXP3 reduction, and MFD was included to allow for not being able to reach the MTD.

The secondary objectives were to determine the preliminary antitumor activity of AZD8701 when administered as monotherapy or in combination with durvalumab. PD, measured as the posttreatment change from baseline *FOXP3* mRNA expression levels in tumor tissue, and PK with AZD8701 as monotherapy and in combination with durvalumab were also predefined secondary objectives. A prespecified statistical analysis plan was in place for the PD assessment, and the subgroup of patients who agreed to participate in these analyses provided informed consent for mandatory biopsies.

### Assessments

#### Safety

All AE and serious AE (SAE), including DLT, were graded according to NCI CTCAE version 5 and classified according to the Medical Dictionary for Regulatory Activities version 26. A DLT was defined as any AZD8701- or AZD8701/durvalumab-related grade ≥3 toxicity (not clearly attributable to the disease, other concomitant medications, disease-related processes under investigation, or another non–drug-related etiology), with specified modifications or exceptions (Supplementary Material S1) occurring from the first dose of AZD8701 until the end of the first 28-day cycle of dose-escalation parts 1 and 3. Blood samples for the assessment of clinical chemistry, including liver function and hematology, were taken during screening on days 1, 2, 3, 5, 8, 15, and 22 of cycle 1, and days 1, 8, 15, and 22 of subsequent cycles.

#### Efficacy

Objective response rate, disease control rate at 16 weeks, time to response, duration of response, and percentage change in tumor size were assessed using RECIST version 1.1, based on central readings of magnetic resonance imaging or computer tomography scans performed at baseline on day 1 of cycle 3, then every 8 weeks (±7 days) until week 48, then every 12 weeks (±7 days) until disease progression, and at the end of treatment. The same imaging technique was used in all individual patients. Progression-free survival was defined as the time from the start of treatment until the date of objective disease progression or death (whichever occurred first). Patients without disease progression or who had died at the time of analysis were censored at the most recent date of assessment. Overall survival (OS) was defined as the time from the start of treatment until the date of death from any cause. Patients alive at the data cutoff were censored on the most recent date when known to be alive. The OS rate at 18 months and the associated 95% confidence interval (CI) were determined using the Kaplan–Meier method.

#### PK

Venous blood samples for PK analysis of AZD8701 were taken preinfusion and at 0.5, 1, 1.5, 2, 4, 7, and 24 hours postinfusion, starting on day 1 of cycle 1 and day 22 of cycle 2. Preinfusion samples were taken on days 3, 5, and 8 of cycle 1 and day 1 of cycles 2, 3, and 4, and a further sample was taken 105 days after the last dose of AZD8701.

Urine samples were collected from 0 to 7 and 7 to 24 hours after AZD8701 infusion started on day 1 of cycle 1 and day 22 of cycle 2.

AZD8701 (plasma and urine) and durvalumab (serum) concentrations were determined using high-performance liquid chromatography–tandem mass spectrometry and electrochemiluminescence sandwich immunoassay, respectively, using validated bioanalytic methods. AZD8701 PK analyses were performed by noncompartmental analysis using Phoenix WinNonlin (Certara RRID: SCR_024504).

#### PD

Mandatory fresh tumor biopsy samples were collected from patients in the PD analysis parts (parts 1B and C) at screening and on day 1 of cycle 2 (±7 days) to analyze the percentage change in *FOXP3* mRNA expression. In addition, tumor biopsies could be collected from patients in the dose-escalation parts (parts 1A and 3A) on day 1 of cycle 2 and from patients in any study part at disease progression if consent was provided. Where clinically feasible, fresh tumor samples were collected using four core image-guided needle biopsies. Tumor biopsy samples were collected from nonbone lesions of individuals with bone metastases.

Whole blood samples for the analysis of gene expression, including *FOXP3,* were collected from all patients at screening, days 1 and 15 of cycle 1, day 1 of cycle 2, all subsequent cycles, and at progression. RNA sequencing was performed by the Almac Group on blood samples collected in PAXgene blood RNA tubes.

### RNA sequencing and data processing

RNA isolation and sequencing were performed by the Almac Group. Briefly, RNA was extracted from fresh frozen tumor and blood samples. Libraries were constructed using the Roche KAPA HyperPrep kit and sequenced (Illumina) at a depth of 100 million base reads (for tissue: 150 bp paired-end reads; for blood: 75 bp paired-end reads).

Sequencing reads were processed and quality-checked using the pipeline implemented in bcbio-nextgen [version 1.2.9; RRID SCR_004316 (26)]. The reads were aligned to the UCSC build GRCh38 *Homo sapiens* genome using STAR [version 2 6.1; RRID: SCR_005622 (27)]. Alignments were evaluated for evenness of coverage, rRNA content, genomic context, and complexity using a range of bespoke and publicly available tools (FastQC, RRID: SCR_014583; Qualimap, RRID: SCR_001209; ref. 28). Transcripts per million measurements were generated by Salmon alignment–based quantification (29) and used to estimate the abundance of the *FOXP3* gene. Batch effects were adjusted using ComBat-seq (30).

### T, B, and natural killer cell assay and proliferating T-cell assay

Flow cytometry panels were used to detect T, B, NK cells, and proliferating T cells in human whole blood samples taken at the same times as those noted for the PD analysis. A detailed description of the T, B, and NK cell assay and proliferating T-cell assays can be found in Supplementary Material S1. The T, B, and NK cell panels of the assays were used to calculate the T-cell fraction, which was then used to calculate the percentage of proliferating T cells.

### miRNAscope *in situ* hybridization and HALO image analysis

ISH signal density (ASO dots/mm^2^) in the tumor and stroma regions of the tissue was quantified, as described in Supplementary Material S1.

### Statistical analysis

The number of patients enrolled was determined based on the number required to obtain adequate tolerability, safety, and PK data while exposing as few patients as possible to AZD8701; power calculations were not done for this phase I study because of the limited sample size and heterogeneous patient population. For data analysis, categorical data were summarized by frequency distribution and continuous variables were summarized by descriptive statistics. Unless stated otherwise, two-sided CI were calculated at 95% confidence. The definitions of the analysis sets used are presented in Supplementary Table S1.

The potential correlation between changes in FOXP3 expression in the blood and tumors was determined using Pearson’s correlation.

### Data availability

Data underlying the findings described in this article may be obtained in accordance with AstraZeneca’s data sharing policy padescribed at https://astrazenecagrouptrials.pharmacm.com/ST/Submission/Disclosure. Data for studies directly listed on paVivli can be requested through Vivli at www.vivli.org. Data for studies not listed on Vivli can be requested through Vivli at https://vivli.org/members/enquiries-about-studies-not-listed-on-the-vivli-platform/. An AstraZeneca Vivli member page is also available outlining further details: https://vivli.org/ourmember/astrazeneca/.

## Results

### Baseline characteristics and disposition

A total of 63 patients were enrolled, 45 of whom were treated with AZD8701 monotherapy and 18 with AZD8701 and durvalumab. The baseline patient and disease characteristics are shown in Table 1 (the representativeness of study participants is described in Supplementary Table S2). Most patients were heavily pretreated, with a median number (range) of 3 (1–8) previous systemic regimens in the monotherapy cohort and a median of 2.5 (1–5) previous systemic regimens in the combination therapy cohort. The number of patients treated at each dose in the dose-escalation and PD parts of the monotherapy cohort is shown in Supplementary Fig. S1B.

**Table 1.** Baseline patient and disease characteristics (safety population).

	Monotherapy							Combination therapy			
Characteristic	60 mg	120 mg	240 mg	480 mg	720 mg	960 mg	Total	240 mg	480 mg	720 mg	Total
	(*n =* 1)	(*n =* 1)	(*n =* 10)	(*n =* 11)	(*n =* 14)	(*n =* 8)	(*n* = 45)	(*n =* 6)	(*n =* 6)	(*n =* 6)	(*n =* 18)
Median age, years (range)	61 (–)	70 (–)	60.5 (35–75)	56 (31–78)	61 (46–73)	51.5 (32–74)	57.8 (31–78)	61 (50–72)	59 (54–77)	53.5 (43–69)	59.0 (43−77)
Sex, female/male (*n*)	0/1	1/0	9/1	5/6	3/11	3/5	21/24	3/3	5/1	2/4	10/18
Race, *n*											
Asian	0	0	1	0	0	0	1	1	1	0	2
Black or African American	0	0	2	0	0	0	2	0	0	0	0
White	1	1	4	9	10	4	29	4	3	4	11
Other	0	0	3	2	4	4	13	1	2	2	5
Ethnicity, *n*											
Hispanic or Latino	0	0	0	1	0	1	2	0	0	0	0
Not Hispanic or Latino	1	1	7	8	10	4	31	5	4	4	13
Missing	0	0	3	2	4	3	12	1	2	2	5
ECOG PS, *n*											
0	1	0	3	3	7	3	17	3	3	5	11
1	0	1	7	8	7	5	28	3	3	1	7
Site of cancer at study entry, *n*											
Bone	0	0	2	4	1	1	8	1	1	2	4
Brain	0	0	0	0	0	0	0	1	0	0	1
Distant lymph nodes	1	1	6	4	7	1	20	3	4	2	9
Local lymph nodes	0	0	4	2	6	3	15	6	3	1	10
Liver	0	0	3	6	3	4	16	3	3	1	7
Spleen	0	0	0	1	1	1	3	0	0	0	0
Other visceral organs	0	0	2	3	2	4	3	3	1	3	7
Median number of prior systemic therapy regimens, *n* (range)	2 (–)	8 (–)	3 (1–8)	3 (2–8)	3 (1–6)	3 (1–7)	3 (1–8)	4 (1–5)	3 (2–5)	1 (1–2)	2.5 (1–5)
Best response to a most recent line of therapy, *n*											
CR	0	0	0	0	0	0	0	0	0	0	0
PR	0	0	1	2	2	1	6	0	1	4	5
SD	0	0	0	4	4	3	11	2	1	1	4
PD	0	1	4	1	3	3	12	2	3	1	6
NE	1	0	0	0	0	0	1	0	0	0	0
Unknown	0	0	5	2	5	1	13	2	1	0	3
Baseline tumor biomarker status, *n*											
PD-(L)1 positive	0	0	1	1	4	1	7	2	0	0	2
PD-(L)1 negative	1	1	3	1	2	0	8	0	0	2	2
Unknown	0	0	6	9	8	7	30	4	6	4	14

Abbreviations: CR, complete response; ECOG PS, Eastern Cooperative Oncology Group performance status; NE, not evaluable; PD, progressive disease.

All 63 patients received at least one dose of the study therapy. The median (range) duration of exposure to AZD8701 was 1.85 (0.3–17.5) months for patients in the AZD8701 monotherapy cohort and 1.31 (0.5–13.9) months for patients in the combination therapy cohort. The median duration of exposure to durvalumab in the combination therapy cohort was 1.88 (0.9–14.4) months. All patients treated with AZD8701 monotherapy or combination therapy had discontinued treatment at the data cutoff, with progressive disease being the main reason for discontinuation. Of the 45 patients treated with AZD8701 monotherapy, 37 (82.2%) discontinued because of disease progression, three (6.7%) because of AE, three (6.7%) because of investigator decision, one (2.2%) because of patient decision, and one (2.2%) for other reasons; of the 18 patients treated with combination therapy, 15 (83.3%) discontinued because of disease progression, two (11.1%) because of an AE, and two (5.6%) for other reasons.

Eight patients were lost to follow-up: six in the monotherapy cohort and two in the combination therapy cohort. Per protocol, patients were considered lost to follow-up if they could not be contacted by the study site. The median (range) potential duration of follow-up was 17.76 (1.8–34.3) months in the AZD8701 monotherapy cohort and 13.44 (1.8–19.4) months in the combination therapy cohort.

### Safety

#### AZD8701 monotherapy

All patients who received AZD8701 monotherapy experienced at least one treatment-emergent AE (TEAE), and 82.2% experienced at least one AZD8701-related TEAE (Table 2). The most frequently observed TEAE across all dose cohorts were anemia (*n* = 18, 40.0%), fatigue (*n =* 16, 35.6%), diarrhea (*n =* 15, 33.3%), asthenia (*n =* 14, 31.1%), pyrexia (*n =* 12, 26.7%), increased alanine aminotransferase (ALT; *n =* 11, 24.4%), increased aspartate aminotransferase (AST), and increased blood creatinine (*n =* 10, 22.2%, each; Supplementary Table S3). Grade ≥3 TEAE occurred in 27 patients (60.0%), the most common of which were anemia (*n =* 5, 11.1%), increased γ-glutamyltransferase (*n =* 4, 8.9%), abdominal pain, increased ALT, and hypokalemia (*n =* 3, 6.7% each; Supplementary Table S3). Treatment-emergent SAE were observed in 13 patients (28.9%; Table 2), with the following SAE occurring in two patients (4.4%) each: pyrexia resulting in hospitalization, one each at 480 mg (grade 2, not related to AZD8701) and 720 mg (grade 1, related to AZD8701); grade 3 increased ALT related to AZD8701 treatment, both at 960 mg; and grade 3 pulmonary embolism not related to AZD8701 treatment, both at 720 mg. AE that required treatment discontinuation occurred in three patients: one patient at 480 mg (spinal cord compression not related to AZD8701 after cycle 2, day 15) and two at 960 mg (AZD8701-related increased ALT after cycle 1, day 15 and cycle 1, day 22, respectively).

**Table 2.** Safety summary.

	Monotherapy							Combination therapy			
Characteristic	60 mg	120 mg	240 mg	480 mg	720 mg	960 mg	Total	240 mg	480 mg	720 mg	Total
	(*n* = 1)	(*n* = 1)	(*n* = 10)	(*n* = 11)	(*n* = 14)	(*n* = 8)	(*n* = 45)	(*n* = 6)	(*n* = 6)	(*n* = 6)	(*n* = 18)
Any AE, *n* (%)	1 (100)	1 (100)	10 (100)	11 (100)	14 (100)	8 (100)	45 (100)	6 (100)	6 (100)	6 (100)	18 (100)
Any grade 3/4 AE, *n* (%)	0	0	5 (50)	5 (45.5)	10 (71.4)	7 (87.5)	27 (60.0)	4 (66.7)	4 (66.7)	5 (83.3)	13 (72.2)
Any SAE, *n* (%)^a^	0	0	2 (20)	2 (18.2)	6 (42.9)	3 (37.5)	13 (28.9)	1 (16.7)	3 (50.0)	3 (50.0)	7 (38.9)
Any DLT, *n* (%)	0	0	0	0	0	1 (12.5)	1 (2.2)	0	0	0	0
Any DLT-like event, *n* (%)^b^	0	0	0	0	0	2 (25.0)	2 (4.4)	0	0	0	0
Death, *n* (%)	0	0	0	0	0	0	0	0	0	0	0
Any AE related to AZD8701 or durvalumab, *n* (%)											
Related to AZD8701	1 (100)	1 (100)	8 (80.0)	9 (81.8)	13 (92.9)	5 (62.5)	37 (82.2)	4 (66.7)	4 (66.7)	6 (100)	14 (77.8)
Related to durvalumab	—	—	—	—	—	—	—	3 (50.0)	4 (66.7)	5 (83.3)	12 (66.7)
Any SAE related to AZD8701 or durvalumab, *n* (%)^a^											
Related to AZD8701	0	0	0	0	2 (14.3)	2 (25.0)	4 (8.9)	0	0	1 (16.7)	1 (5.6)
Related to durvalumab	—	—	—	—	—	—	—	0	0	1 (16.7)	1 (5.6)

a SAE met the following criteria: death, life-threatening, required inpatient hospitalization, prolongation of existing hospitalization, persistent or significant disability/incapacity, important medical event, or congenital anomaly/birth defect (in the offspring of the subject).

b Any AE meeting the DLT criteria but occurring outside of the DLT evaluation period could be defined as a DLT-like event after consultation with the sponsor and investigators.

The most frequently observed AZD8701-related AE of any grade are shown in Supplementary Table S4. Notably, AZD8701-related increased ALT and AST occurred only in patients treated with AZD8701 480 mg or higher and were most frequent at the 960 mg dose (*n =* 5, 62.5% and *n =* 4, 50.0% for increased ALT and increased AST, respectively; Fig. 1A). Grade ≥3 AZD8701-related AE occurred in seven patients (15.6%), with increased ALT being the only AZD8701-related grade ≥3 AE experienced by more than one patient (*n =* 3, 6.7%). Other grade ≥3 AZD8701-related AE included anemia, lymphopenia, increased AST, increased γ-glutamyltransferase, increased lipase, and transverse myelitis (*n =* 1, 2.2% each). Transverse myelitis, which was also an SAE, was treated with methylprednisolone or prednisone and resolved by the time of data cut-off. AZD8701-related SAE occurred in four patients (8.9%; Table 2; AZD8701 720 mg, pyrexia and transverse myelitis; AZD8701 960 mg, increased ALT and AST).

#### AZD8701 and durvalumab combination therapy

All patients receiving combination therapy experienced at least one TEAE (Table 2). The most frequently observed TEAE across all doses were anemia (*n =* 9, 50%), pyrexia (*n =* 6, 33.3%), and diarrhea, dyspnea, fatigue, nausea, vomiting, and constipation (*n =* 5, 27.8% each; Supplementary Table S5). Grade ≥3 TEAE observed in more than one patient were anemia (*n =* 3, 16.7%), increased ALT, and lymphopenia (both *n =* 2, 11.1%). Treatment-emergent SAE were observed in seven patients (38.9%). One patient at 720 mg developed grade 2 renal failure related to AZD8701 and durvalumab. The patient presented with grade 1 increased creatinine levels at screening, which increased to grade 2 during cycle 1. Treatment with oral corticosteroids led to stabilization of creatinine levels, but AZD8701 was permanently discontinued after cycle 1, day 22; durvalumab was continued after a delay, and the event was reported to be not resolved at the end of the study. Another patient treated with this AZD8701 dose who had type 2 diabetes mellitus experienced grade 2 renal impairment also related to AZD8701 and durvalumab, leading to permanent discontinuation of AZD8701 after cycle 1, day 8 and durvalumab after cycle 1, day 1. The event was reported to be resolved at the end of the study.

Most patients experienced at least one AE related to AZD8701 (*n =* 14, 77.8%) or durvalumab (*n =* 12, 66.7%, Table 2). AZD8701- and durvalumab-related AE observed in >10% of patients are shown in Supplementary Tables S6 and S7, respectively. Grade ≥3 AE related to AZD8701 included increased ALT, lymphopenia (both *n =* 2, 11.1%), fatigue, and increased AST (both *n =* 1, 5.6%). Grade ≥3 AE related to durvalumab were lymphopenia, increased ALT (both *n =* 2, 11.1%), and fatigue (*n =* 1, 5.6%). One patient at 720 mg experienced SAE of increased ALT and AST that were judged to be related to both AZD8701 and durvalumab.

#### DLT

One patient treated with AZD8701 960-mg monotherapy experienced DLT with increased ALT (grade 3). No DLT were observed in patients treated with AZD8701 combined with durvalumab.

#### Death

During the study, 37 patients (58.7%) died, none while on treatment, and none of the deaths were related to AE. The most common cause of death was the disease under investigation (*n =* 34, 91.9%).

#### Liver function parameters

As shown in Fig. 1, the levels of alkaline phosphatase, ALT, AST, and bilirubin tended to increase over time with both AZD8701 monotherapy and combination therapy. Moreover, a shift from normal levels of alkaline phosphatase, ALT, and/or AST at baseline to levels above the upper limit of normal posttreatment was recorded in 30% to 60% of the patients (Supplementary Tables S8 and S9).

Coagulation tests, albumin, and protein levels remained largely unchanged when increases in transaminase levels were reported (Supplementary Tables S8 and S9).

### Dose modifications

#### Dose modifications in the AZD8701 monotherapy arm

Two patients (4.4%) had AZD8701 dose delays, both affecting one dose, with delays of 4 days at AZD8701 480 mg and 26 days for AZD8701 720 mg. In addition, there were three single-infusion interruptions (6.7% of patients), one each in the 240-, 720-, and 960-mg dose cohorts. All three patients received the entire planned dose, and none of the dose interruptions were related to AE. Dose omissions occurred in 28 patients (62%) treated with AZD8701 monotherapy, with a median (range) of two (1–13) omitted doses per patient. Dose omissions occurred in all dose cohorts, except for AZD8701 120 mg. AE were the reason for dose omission in 20/28 patients. AZD8701 dose reduction occurred only in the 720-mg cohort, in which a single AZD8701 dose reduction due to an AE occurred in two patients (14.3%).

#### Dose modifications in the AZD8701 + durvalumab combination therapy arm

Three AZD8701 doses were delayed in two patients in the 480-mg cohort, and one dose was delayed in a single patient in the 720-mg cohort; three of these delays were due to AE. At least one AZD8701 dose was omitted in most patients (94.4%) across all dose cohorts, with a median (range) number of omitted doses per patient of three (1–43). AE were responsible for 83.3% of all dose omissions. AZD8701 dose reduction occurred only in the 720-mg cohort: two patients had a dose reduction, one of which was due to an AE.

Durvalumab dose delays occurred in two patients in the 480-mg cohort (33.3%) and in one patient in the 720-mg dose cohort, all due to AE. Durvalumab doses were omitted in five patients (27.8%): two in the AZD8701 240-mg cohort, one in the AZD8701 480-mg cohort, and two in the AZD8701 720-mg cohort. AE were the reason for omissions in four patients. No durvalumab dose reduction was reported.

### Efficacy

**Figure 2.**
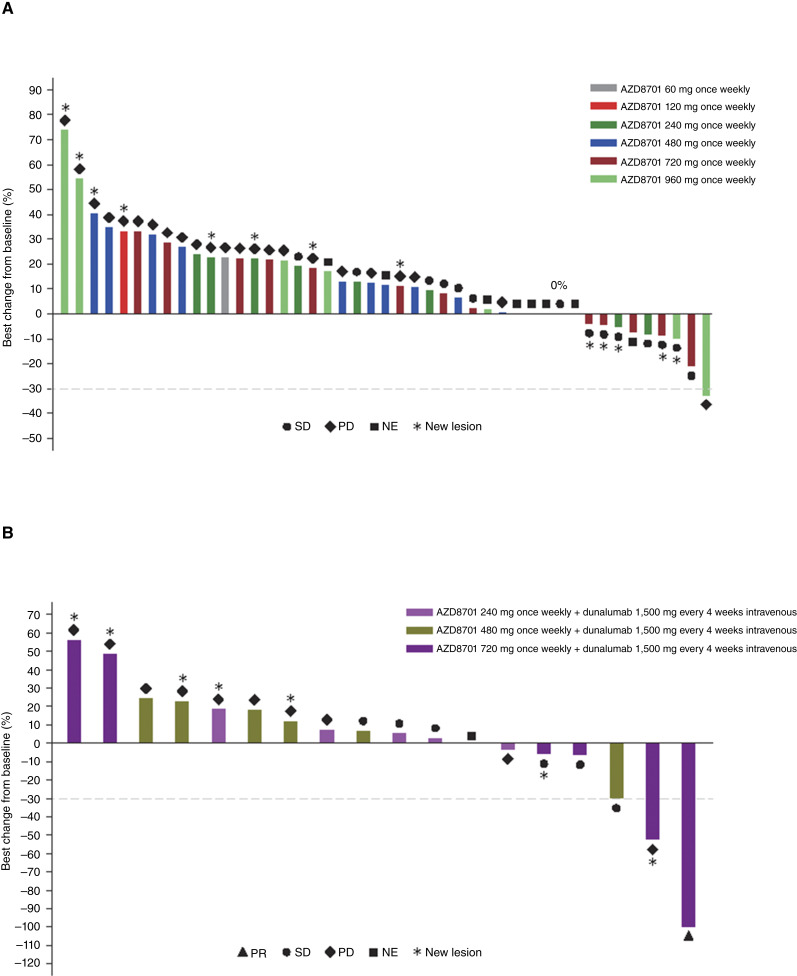
Waterfall plot showing the best overall response for (**A**) AZD8701 monotherapy and (**B**) AZD8701 in combination with durvalumab. NE, nonevaluable; PD, progressive disease.

The response data are shown in Supplementary Table S10. No objective responses were observed in patients treated with AZD8701 monotherapy. However, 26.7% and 15.6% of these patients had stable disease (SD) for ≥16 and ≥24 weeks, respectively (Fig. 2A). One patient treated with AZD8701 720 mg and durvalumab had a confirmed partial response (PR) after 5.6 months. This patient had cervical cancer that had previously been treated with carboplatin/paclitaxel and atezolizumab/bevacizumab or placebo/cisplatin/gemcitabine and received 1.0 month of AZD8701 and 7.2 months of durvalumab, with a PR after five treatment cycles. The patient permanently discontinued AZD8701 because of renal failure. Another patient treated with AZD8701 and durvalumab showed an unconfirmed PR. This patient had bladder cancer that had previously been treated with carboplatin/gemcitabine and atezolizumab/cabozantinib and received AZD8701 480 mg for 4.9 months and durvalumab for 5.4 months, responding at disease assessment 2 (day 1, cycle 4). Moreover, 22.2% and 16.7% of the patients who received the combination of AZD8701 and durvalumab had SD for ≥16 and ≥24 weeks, respectively (Fig. 2B).

The median progression-free survival was 1.8 months (95% CI, 1.7–2.0) and 1.9 months (95% CI, 1.5–5.6) in patients treated with AZD8701 alone and with AZD8701 and durvalumab, respectively. The median OS was 11.3 months (95% CI, 8.0–13.4) and 12.2 months (95% CI, 4.2–not evaluable), respectively.

### AZD8701 PK

**Figure 3.**
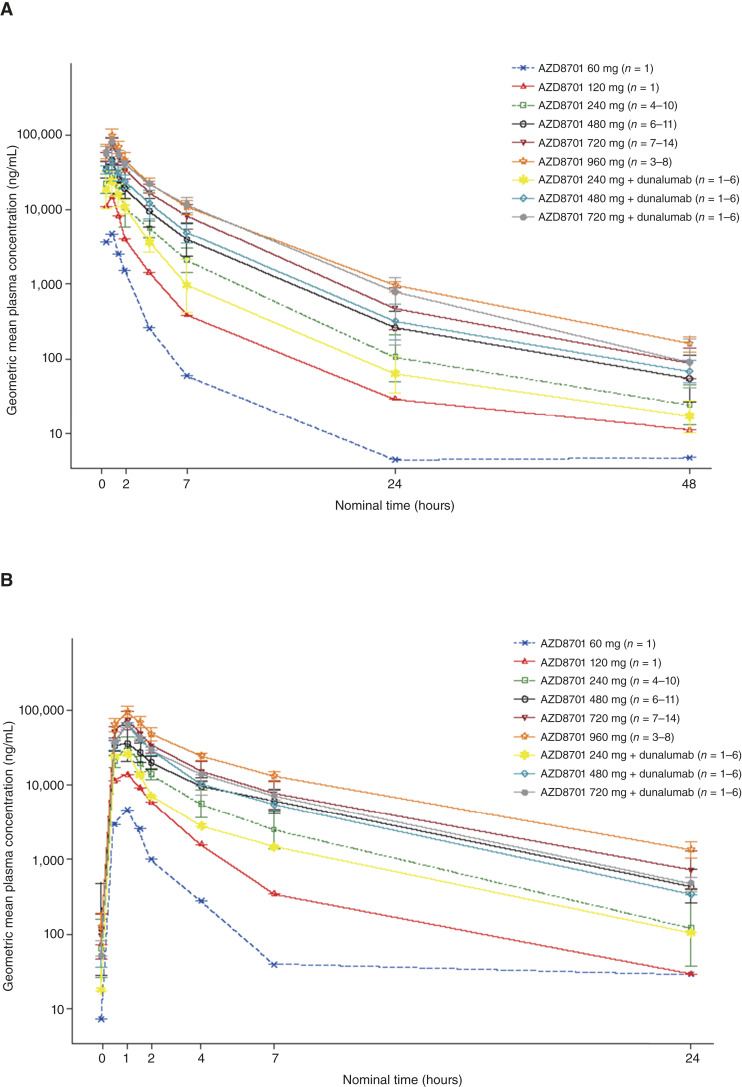
Geometric mean (± geometric SD) plasma concentration–time profiles of AZD8701 following single (**A**) and multiple (**B**) doses of AZD8701 60 to 960 mg alone or in combination with durvalumab (semi-log scale).

AZD8701 plasma concentrations decreased in a multiphasic manner, with a rapid distribution phase followed by a slower elimination phase. AZD8701 exposure generally seemed to increase proportionally with the dose. Minimal accumulation of AZD8701 was observed following multiple doses. In general, the single- and multiple-dose PK of AZD8701 when administered with durvalumab (part 3) were similar to those of AZD8701 administered alone (Part 1; Fig. 3; Supplementary Table S11). The excretion of AZD8701 in urine was low following both single and multiple doses, and renal clearance represented a minor fraction of the total plasma clearance (data not shown). The geometric mean Ctroughss (4 weeks post-first dose) at doses ≥240 mg exceeded the Ctroughss in mouse tumor models, suggesting that the systemic concentrations of AZD8701 doses explored were consistent with those associated with tumor growth inhibition in mice (50 mg/kg twice weekly; ref. 17).

### PD

**Figure 4.**
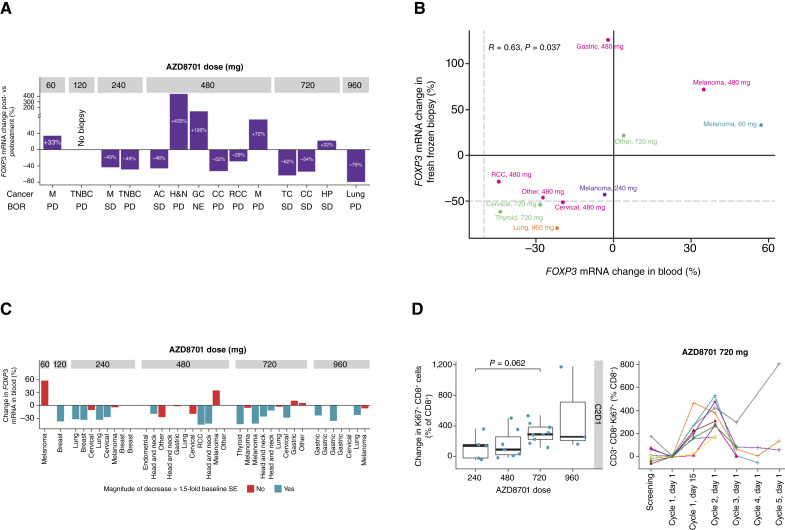
Pharmacodynamic effects at cycle 2, day 1. **A,** Changes in *FOXP3* mRNA expression from baseline in on-treatment biopsy samples. **B,** Correlation between changes in *FOXP3* expression in tumor and in blood for patients with paired biopsy samples. **C,** Changes in *FOXP3* expression in the blood of all patients with available data. **D,** Changes in Ki67^+^ CD8^+^ T cells in the periphery. AC, adrenal carcinoma; BOR, best overall response; CC, cervical cancer; GC, gastric cancer; H&N, head and neck carcinoma; HP, hemangiopericytoma; M, melanoma; NE, not evaluable; PD, progressive disease; TC, thyroid cancer; TNBC, triple-negative breast cancer.

Changes in *FOXP3* mRNA expression levels from baseline in tumor samples were available for 13 patients treated with AZD8701 monotherapy. The median (range) change in *FOXP3* mRNA expression levels was 25.6% (not applicable) with AZD8701 60 mg, −34.8% (−49.9 to −19.7) with 240 mg, −5.5% (−39.3 to 413.2) with 480 mg, −28.6% (−42.0 to 28.5) with 720 mg, and −67.0% (NA) in patients treated with AZD8701 960 mg. Overall, reductions in *FOXP3* mRNA expression levels of −29% to −79% were observed in 8/13 (61.5%) patients treated with AZD8701 monotherapy for whom data were available (Fig. 4A), suggesting on-target ASO activity. However, the extent of knockdown was heterogeneous, and there was no clear dose-dependent relationship (Fig. 4A). Furthermore, there was no apparent relationship between the extent of *FOXP3* knockdown and tumor shrinkage (data not shown). *In situ* hybridization staining for ASO was performed in the subset of six patients with available samples; these patients were treated with doses of 240 to 960 mg. This revealed the presence of the ASO in the tumor epithelium in four out of six cases and the stroma in five out of six cases, indicating delivery of the ASO to the tumor (Supplementary Fig. S2).

A significant correlation between *FOXP3* knockdown in the tumor and in the blood at cycle 2, day 1, was observed for patients with a paired biopsy sample (*R* = 0.63; *P* < 0.05; Fig. 4B), supporting on-target ASO activity in both the tumor and periphery. Based on this, the change in *FOXP3* expression in peripheral blood was examined in all patients with available peripheral data rather than only in patients with paired biopsy samples. A reduction in *FOXP3* mRNA in the blood on cycle 2, day 1 of ≥1.5-fold the baseline SE was observed in 16/28 patients (57%; Fig. 4C), suggesting that the extent of *FOXP3* knockdown in the periphery was also heterogeneous.

A significant trend toward an increase in Ki67^+^CD8^+^ T cells in the periphery on cycle 2, day 1 with increased AZD8701 dose was noted, indicating potentially enhanced activation of cytotoxic T cells. This immune response tended to peak on cycle 2, day 1 (Fig. 4D).

## Discussion

New immunotherapeutic approaches are needed to improve outcomes in patients who do not benefit from long-term PD-(L)1 inhibition. We describe the first in-human study of AZD8701, a novel antisense technology targeting the immunosuppressive function of Tregs in the TME by reducing Treg *FOXP3* mRNA expression.

This study demonstrated that AZD8701 dose escalation up to 720 mg as monotherapy and in combination with durvalumab was well-tolerated by patients with advanced solid tumors. TEAE were generally manageable in patients treated with AZD8701 as monotherapy or in combination with durvalumab. Notably, asymptomatic liver function test abnormalities, such as increased ALT and AST, limited further dose escalation and were recognized as dose-dependent. Management of such toxicities, including treatment interruption within the first two cycles and, in some cases, dose reduction, resulted in recovery to normal transaminase levels within a few weeks. Historically, ASO as a class have been associated with hepatotoxicity, renal toxicity, hypersensitivity reactions, and other unique AE (31). Coagulation and protein tests (see Supplementary Tables S8 and S9) indicated that elevated liver enzyme levels were not associated with liver impairment.

Based on preclinical data and modeling, we predicted that higher ASO doses would achieve a greater reduction of FOXP3 protein, although, at the highest protocol-defined dose (960 mg), we expected to achieve a <50% reduction in FOXP3 protein. This meant that in addition to the OBD, we included establishing the MTD or MFD in the primary objective, with the MFD included to allow for not being able to reach the MTD either because of infusion volume or manufacturing constraints. In the absence of significant efficacy with AZD8701 monotherapy and the occurrence of dose-dependent asymptomatic liver function test abnormalities, dose escalation was terminated before these were established.

A potential safety risk when targeting FOXP3 expression in Tregs is the link between *FOXP3* loss-of-function mutations and autoimmune disorders in humans (18). However, apart from one case of transverse myelitis, there was no evidence that the FOXP3 knockdown effect in this study affected self-tolerance at any of the AZD8701 doses tested.

Although AZD8701 may have contributed to durable SD, there was no evidence of a clinical response to AZD8701 when administered as monotherapy. When administered in combination with durvalumab, a confirmed partial tumor response was observed in one patient. Based on these efficacy data, which suggest that AZD8701 has limited activity and safety data, the planned dose-expansion parts of the study were not initiated.

The low objective response rate using the RECIST reported in the present study may be, in part, because the patient population was heavily pretreated. Furthermore, we included patients with multiple tumor types who were not selected for the expression of potential biomarkers of response to AZD8701 or durvalumab because of the lack of validated predictive biomarkers for Treg regulation in the TME. We did, however, try to focus recruitment on patients with tumor types that are known to be sensitive to immunotherapy and have been shown to have generally high Treg infiltration (20–24). However, the heterogeneity of the underlying disease resulting from the lack of validated predictive biomarkers could also have affected the response rates.

It is known that efficacy tends to be reduced compared with earlier lines of therapy in patients treated later (32, 33) and/or after immunotherapy (34, 35), even when active agents are used. In this study, patients treated with AZD8701 or combination therapy received a median of 3.0 and 2.5 previous systemic regimens, respectively. Therefore, testing AZD8701 in earlier disease settings may be warranted. Moreover, combining AZD8701 with nonimmunotherapeutic treatments such as chemotherapy may be worth exploring as an option for patients who have relapsed after immunotherapy.

The efficacy of AZD8701 could, in theory, also be limited by the reduced delivery of ASO to the TME (36). However, using an *in situ* hybridization assay, we directly observed the presence of ASO in the tumor epithelium and stroma; it should be noted that samples for this analysis were available only for a small subset of patients, which limits data interpretation. This means that it is difficult to draw a definitive conclusion on whether ASO delivery to the TME is sufficient to enable robust knockdown, and strategies to improve ASO delivery could be explored. Examples of such strategies include liposomal delivery or conjugates, such as peptides or antibodies, to promote distribution and enhance cellular uptake (36). The assumption that improved tumor exposure might lead to greater efficacy of AZD8701 is supported by the immunological effects observed in the periphery, specifically the increased levels of Ki67^+^ CD8 T cells in the blood (Fig. 4D), which indicate T-cell activation. However, at present, we cannot formally exclude the nonspecific effects of ASO.

Patients with brain metastases who had been treated and were radiographically stable at study entry were eligible to participate in the study; this inclusion criterion was designed to increase inclusiveness to clinical trials. Although ASO generally do not cross the blood–brain barrier, it is recognized that the blood–brain barrier is disrupted in patients with brain tumors, allowing large molecules to cross it. For example, the benefit of immunotherapy in this setting has been documented (37, 38). However, only one patient with stable brain metastases was included in the trial and treated with AZD8701 and durvalumab, so any effect on treatment benefit cannot be determined.

We analyzed changes in *FOXP3* mRNA in baseline and on-treatment tumor samples as a predefined secondary endpoint based on a strong scientific rationale for identifying the biological activity of an ASO directed against *FOXP3*. This is in line with the ASCO Ethical Framework for Including Research Biopsies in Oncology Clinical Trials (39). We demonstrated that AZD8701 as monotherapy causes modest downregulation of *FOXP3* expression in the TME (decreases of 29%–79% in 8/13 patients), thus confirming the FOXP3 knockdown effect observed in preclinical studies (17). However, the knockdown effect was heterogeneous within doses, was not associated with the degree of tumor shrinkage, and a clinically meaningful level of decrease in *FOXP3* expression has not been established. Due to the lack of association between change in FOXP3 expression and AZD8701 dose or tumor shrinkage, it is not possible to draw any definitive conclusions about the significance of the observed changes. However, we hypothesize that other types of immunosuppressive activity could be active in the TME, including myeloid cells or tumor-associated macrophages. Furthermore, ideally, we would have selected patients using biomarkers to identify patients who had had a preexisting antitumor immune response and subsequent Treg-driven immune evasion. Dual selection based on CD8/Treg abundance would be useful in this regard, but better biomarkers are needed.

A strength of the current study design was the clear rationale for AZD8701 dosing: the starting dose was based on the no-observed-adverse-effect level observed in monkeys, and dose escalation followed an accelerated titration design, allowing for single-patient cohorts at AZD8701 doses of 60 and 120 mg, reducing the number of patients who needed to be exposed to AZD8701 during the dose escalation and safety evaluation period. We know from studies of other ASO (40, 41) that doses below 200 mg are well tolerated and have a limited impact on the target, but we included the criterion that if ≥1 NCI CTCAE grade ≥2 drug-related toxicity or DLT occurred in any cohort, the cohort would be expanded to a minimum of three patients. Based on the safety data, single patients were treated with AZD8701 60 and 120 mg, and the dose was then escalated to the first dose of AZD8701 (240 mg), predicted to be in the active clinical dose range of 160 to 660 mg. The use of AZD8701 loading doses followed by weekly dosing in all cohorts was based on preclinical data and data from clinical trials of other agents (40, 41), demonstrating that loading doses are important to ensure that steady-state tissue distributions are reached as rapidly as possible.

In conclusion, we demonstrated that AZD8701 caused heterogeneous knockdown of *FOXP3* in patients with advanced solid tumors. AE were generally manageable, and dose escalation was possible, demonstrating the feasibility of using ASO therapy in the clinical setting. Liver function test abnormalities were common and are likely a class effect of generation 2.5 ASO therapy. Although durable SD was observed in some patients who received AZD8701 monotherapy, and one confirmed partial clinical tumor response was observed in a patient who received AZD8701 in combination with durvalumab, the observed level of efficacy in a population of unselected, heavily pretreated patients did not justify initiating the dose-expansion part of this trial.

## Supplementary Material

Supplementary Table S1 Analysis populations assessed in the trial

Supplementary Table S2 Representativeness of the patient population

Supplementary Table S3 Summary of all adverse events observed in patients treated with AZD8701 monotherapy regardless of realtionship to treatment

Supplementary Table S4 Summary of AZD8701-related adverse events observed in patients treated with AZD8701 monotherapy

Supplementary Table S5 Summary of all adverse events observed with AZD8701 + durvalumab combination therapy, regardless of relationship to therapy

Supplementary Table S6 Summary of AZD8701-related adverse events observed in patients treated with AZD8701 + durvalumab

Supplementary Table S7 Summary of durvalumab-related adverse events observed in patients treated with AZD8701 + durvalumab

Supplementary Table S8 Change of liver parameters relative to normal range in patients treated with AZD8701 monotherapy

Supplementary Table S9 Change of liver parameters relative to normal range in patients treated with AZD8701 + durvalumab

Supplementary Table S10 Detailed response data

Supplementary Table S11 Pharmacokinetic data

Supplementary Figure S1 Shows trial design and patient disposition

Supplementary Figure S2 Shows ASO data and images

Supplementary Material S1 Definition of dose-limiting toxicities (DLT), Inclusion and exclusion criteria, T, B, and natural killer (TBNK) cell assay, Proliferating T cell assay, miRNAscope in situ hybridization and HALO image analysis
