# Supplementary material for: AZD8701, an Antisense Oligonucleotide Targeting FOXP3 mRNA, as Monotherapy and in Combination with Durvalumab: A Phase I Trial in Patients with Advanced Solid Tumors
Source: Clin Cancer Res. 2025 Feb 12;31(8):1449–62. doi: 10.1158/1078-0432.CCR-24-1818 (PMC11995004; doi:10.1158/1078-0432.CCR-24-1818)
Supplement: Supplementary Table S1 — Analysis populations assessed in the trial [file ccr-24-1818_supplementary_table_s1_suppts1.docx]

## Supplementary materials

### Populations for analysis

For purposes of analysis, the study populations were defined as shown in **Supplementary Table S1.**

| **Supplementary Table S1. Populations for analysis** | |
| --- | --- |
| Population/analysis set | Description |
| Enrolled | All patients who signed the informed consent form |
| Safety | All patients who received at least 1 dose of AZD8701 and/or durvalumab |
| PK | All patients who received at least 1 dose of AZD8701 and/or durvalumab and provided at least 1 evaluable PK sample |
| PD | All patients who received at least one dose of AZD8701 and/or durvalumab and provided paired tumor biopsies and peripheral blood samples |
| Evaluable for response | All patients who received at least 1 dose of AZD8701 and/or durvalumab with a baseline imaging tumor assessment |

PD, pharmacodynamic; PK, pharmacokinetics
