# Supplementary material for: AZD8701, an Antisense Oligonucleotide Targeting FOXP3 mRNA, as Monotherapy and in Combination with Durvalumab: A Phase I Trial in Patients with Advanced Solid Tumors
Source: Clin Cancer Res. 2025 Feb 12;31(8):1449–62. doi: 10.1158/1078-0432.CCR-24-1818 (PMC11995004; doi:10.1158/1078-0432.CCR-24-1818)
Supplement: Supplementary Table S2 — Representativeness of the patient population [file ccr-24-1818_supplementary_table_s2_suppts2.docx]

## Supplementary materials

### Representativeness of study participants

**Supplementary Table S2**. Representativeness of study participants.

| **Cancer Type** | Solid tumors |
| --- | --- |
| **Considerations related to:** |  |
| **Sex** | In 2022, the global age-standardised rate (ASR) of all cancers was 212.6/100,000 in men and 186.3/100,000 in women (ratio 1.14).^1^ However, in the USA risk varies with age, and is higher in females than males aged 20–49 years, but higher in males than females aged >75 years.^2^ |
| **Age** | Data for 2022 showed that the global incidence of all cancers increases with age and is highest in patients aged ≥70 years (ASR 1346.7/100,000 in patients aged 70-79 and 1877.4/100,000 in patients aged ≥80 years). The age-related pattern of cancer incidence is similar for most solid tumours.^1^  Median age at diagnosis of cancer is 60–70 years in developed countries.^2,3^ |
| **Race/ethnicity** | Global data on the incidence of cancer by indicate that cancer incidence is higher in White than non-White populations.^4^ In the USA, while analysis is complicated by social factors and over- and underdiagnosis in some groups, ASRs by race range from 295.5/100,000 (Asian/Pacific Islander) to 453.7/100,000 (Black) and 466.6/100,000 (White).^2^ In the UK, ASRs for most cancers are lower in non-White than White ethnicity groups.^5^ |
| **Other considerations** | In 2022, the global ASR of all cancers was 196.9 per 100,000, but varied geographically, from 132.3/100,000 in Africa to 409/100,000 in Oceania, with rates of 280/100,000 in Europe and 364.7/100,000 in North America.^1^  Similarly, ASRs of specific types of cancer also varied geographically both globally and in the USA.^1,2^ |
| **Overall representativeness of this study** | This was a relatively small study that included patients with a wide range of tumor types. Overall, while the median age of the study population in both cohorts and the male:female ratio in the monotherapy cohort were broadly as expected, more males than would have been expected based on the global male:female ASR ratio were enrolled into the combination therapy cohort and the patient population overall was predominantly White. Over-representation in clinical trials of patients who are White has been reported previously.^6^ It seems likely that the low number of patients in this study (N=45 in the monotherapy cohort and 18 in the combination therapy cohort) impacted the potential representativeness of the population. |
