# Supplementary material for: AZD8701, an Antisense Oligonucleotide Targeting FOXP3 mRNA, as Monotherapy and in Combination with Durvalumab: A Phase I Trial in Patients with Advanced Solid Tumors
Source: Clin Cancer Res. 2025 Feb 12;31(8):1449–62. doi: 10.1158/1078-0432.CCR-24-1818 (PMC11995004; doi:10.1158/1078-0432.CCR-24-1818)
Supplement: Supplementary Table S3 — Summary of all adverse events observed in patients treated with AZD8701 monotherapy regardless of realtionship to treatment [file ccr-24-1818_supplementary_table_s3_suppts3.docx]

## Supplementary materials

Supplementary Table S3. Summary of AEs occurring in ≥10% of patients receiving AZD8701 monotherapy.

| AE by preferred term, n (%) | **Any grade**  (*n =* 45) | **Grade ≥3**  (*n =* 45) |
| --- | --- | --- |
| Anemia | 18 (40.0) | 5 (11.1) |
| Fatigue | 16 (35.6) | 0 |
| Diarrhea | 15 (33.3) | 0 |
| Asthenia | 14 (31.1) | 2 (4.4) |
| Pyrexia | 12 (26.7) | 0 |
| ALT increase | 11 (22.2) | 3 (6.7) |
| AST increase | 10 (22.2) | 1 (2.2) |
| Blood creatinine increase | 10 (22.2) | 0 |
| Cough | 9 (20.0) | 0 |
| Pruritus | 8 (17.8) | 0 |
| Arthralgia | 7 (15.6) | 0 |
| Constipation | 7 (15.6) | 1 (2.2) |
| Dyspnea | 7 (15.6) | 0 |
| Headache | 7 (15.6) | 1 (2.2) |
| Nausea | 7 (15.6) | 0 |
| Vomiting | 7 (15.6) | 1 (2.2) |
| Decrease appetite | 6 (13.3) | 0 |
| Chills | 6 (13.3) | 0 |
| Abdominal pain | 5 (11.1) | 3 (6.7) |
| GGT | 5 (11.1) | 4 (8.9) |
| Tumor pain | 5 (11.1) | 1 (2.2) |
| Dizziness | 5 (11.1) | 0 |
| Dehydration | 5 (11.1) | 0 |

AE, adverse event; ALT, alanine aminotransferase; AST, aspartate aminotransferase; GGT, gamma-glutamyl transferase.
