# Supplementary material for: AZD8701, an Antisense Oligonucleotide Targeting FOXP3 mRNA, as Monotherapy and in Combination with Durvalumab: A Phase I Trial in Patients with Advanced Solid Tumors
Source: Clin Cancer Res. 2025 Feb 12;31(8):1449–62. doi: 10.1158/1078-0432.CCR-24-1818 (PMC11995004; doi:10.1158/1078-0432.CCR-24-1818)
Supplement: Supplementary Table S4 — Summary of AZD8701-related adverse events observed in patients treated with AZD8701 monotherapy [file ccr-24-1818_supplementary_table_s4_suppts4.docx]

## Supplementary materials

**Supplementary Table S4.** Summary of AZD8701-related AEs occurring in >10% of patients with AZD8701 monotherapy.

| **AZS8701-related AE, n (%)** | **60 mg**  **(*n =* 1)** | **120 mg**  **(*n =* 1)** | **240 mg**  **(*n =* 10)** | **480 mg**  **(*n =* 11)** | **720 mg**  **(*n =* 14)** | **960 mg**  **(*n =* 8)** | **Total**  **(*n =* 45)** |
| --- | --- | --- | --- | --- | --- | --- | --- |
| Fatigue | 1 (100) | 1 (100) | 3 (30) | 1 (9.1) | 4 (28.6) | 0 | 10 (22.2) |
| Asthenia | 0 | 0 | 0 | 1 (9.1) | 6 (42.9) | 2 (25.0) | 9 (20.0) |
| Pyrexia | 1 (100) | 1 (100) | 3 (30.0) | 1 (9.1) | 3 (21.4) | 0 | 9 (20.0) |
| ALT increase | 0 | 0 | 0 | 1 (9.1) | 3 (21.4) | 5 (62.5) | 9 (20.0) |
| AST increase | 0 | 0 | 0 | 1 (9.1) | 3 (21.4) | 4 (50.0) | 8 (17.8) |
| Anemia | 0 | 1 (100) | 1 (10.0) | 2 (18.2) | 2 (14.3) | 1 (12.5) | 7 (15.6) |
| Diarrhea | 0 | 0 | 3 (30.0) | 2 (18.2) | 2 (14.3) | 0 | 7 (15.6) |
| Pruritus | 0 | 1 (100) | 2 (20.0) | 2 (18.2) | 1 (7.1) | 0 | 6 (13.3) |

AE, adverse event; ALT, alanine aminotransferase; AST, aspartate aminotransferase.
