# Supplementary material for: AZD8701, an Antisense Oligonucleotide Targeting FOXP3 mRNA, as Monotherapy and in Combination with Durvalumab: A Phase I Trial in Patients with Advanced Solid Tumors
Source: Clin Cancer Res. 2025 Feb 12;31(8):1449–62. doi: 10.1158/1078-0432.CCR-24-1818 (PMC11995004; doi:10.1158/1078-0432.CCR-24-1818)
Supplement: Supplementary Table S5 — Summary of all adverse events observed with AZD8701 + durvalumab combination therapy, regardless of relationship to therapy [file ccr-24-1818_supplementary_table_s5_suppts5.docx]

## Supplementary materials

Supplementary Table S5. Summary of AEs occurring in >10% of patients treated with AZD8701 combination therapy.

| AE by preferred term, n (%) | **Any grade**  (*n =* 18) | **Grade ≥3**  (*n =* 18) |
| --- | --- | --- |
| Anemia | 9 (50.0) | 3 (16.7) |
| Pyrexia | 6 (33.3) | 0 |
| Constipation | 5 (27.8) | 0 |
| Diarrhea | 5 (27.8) | 0 |
| Dyspnea | 5 (27.8) | 0 |
| Fatigue | 5 (27.8) | 1 (5.6) |
| Nausea | 5 (27.8) | 0 |
| Vomiting | 5 (27.8) | 0 |
| AST increase | 4 (22.2) | 1 (5.6) |
| Asthenia | 4 (22.2) | 0 |
| Decreased appetite | 4 (22.2) | 0 |
| Pruritus | 4 (22.2) | 0 |
| Thrombocytopenia | 4 (22.2) | 0 |
| Abdominal pain (upper) | 3 (16.7) | 0 |
| ALT increase | 3 (16.7) | 2 (11.1) |
| Blood creatinine increase | 3 (16.7) | 0 |
| Headache | 3 (16.7) | 0 |
| Pneumonia | 3 (16.7) | 1 (5.6) |
| Pulmonary embolism | 3 (16.7) | 0 |
| Back pain | 2 (11.1) | 0 |
| Blood bilirubin increase | 2 (11.1) | 1 (5.6) |
| COVID-19 | 2 (11.1) | 0 |
| Chills | 2 (11.1) | 0 |
| Cough | 2 (11.1) | 0 |
| Embolism | 2 (11.1) | 0 |
| Hypokalemia | 2 (11.1) | 0 |
| Lymphopenia | 2 (11.1) | 2 (11.1) |
| Maculo-papular rash | 2 (11.1) | 0 |
| Pleural effusion | 2 (11.1) | 0 |
| UTI | 2 (11.1) | 1 (5.6) |

AE, adverse event; ALT, alanine aminotransferase; AST, aspartate aminotransferase; UTI, urinary tract infection.
