# Supplementary material for: AZD8701, an Antisense Oligonucleotide Targeting FOXP3 mRNA, as Monotherapy and in Combination with Durvalumab: A Phase I Trial in Patients with Advanced Solid Tumors
Source: Clin Cancer Res. 2025 Feb 12;31(8):1449–62. doi: 10.1158/1078-0432.CCR-24-1818 (PMC11995004; doi:10.1158/1078-0432.CCR-24-1818)
Supplement: Supplementary Table S6 — Summary of AZD8701-related adverse events observed in patients treated with AZD8701 + durvalumab [file ccr-24-1818_supplementary_table_s6_suppts6.docx]

## Supplementary materials

**Supplementary Table S6.** Summary of AZD8701-related AEs occurring in >10% of patients with AZD8701 and durvalumab combination therapy.

| **AZD8701-related AE, n (%)** | **240 mg**  **(*n =* 6)** | **480 mg**  **(*n =* 6)** | **720 mg**  **(*n =* 6)** | **Total**  **(*n =* 18)** |
| --- | --- | --- | --- | --- |
| Nausea | 2 (33.3) | 1 (16.7) | 1 (16.7) | 4 (22.2) |
| Vomiting | 1 (16.7) | 2 (33.3) | 1 (16.7) | 4 (22.2) |
| AST increased | 0 | 2 (33.3) | 2 (33.3) | 4 (22.2) |
| Constipation | 1 (16.7) | 1 (16.7) | 1 (16.7) | 3 (16.7) |
| Diarrhea | 0 | 1 (16.7) | 2 (33.3) | 3 (16.7) |
| Pyrexia | 2 (33.3) | 1 (16.7) | 0 | 3 (16.7) |
| ALT increased | 0 | 2 (33.3) | 1 (16.7) | 3 (16.7) |
| Thrombocytopenia | 0 | 1 (16.7) | 2 (33.3) | 3 (16.7) |
| Blood creatinine increased | 0 | 1 (16.7) | 1 (16.7) | 2 (11.1) |
| Chills | 1 (16.7) | 1 (16.7) | 0 | 2 (11.1) |
| Decreased appetite | 0 | 1 (16.7) | 1 (16.7) | 2 (11.1) |
| Fatigue | 1 (16.7) | 0 | 1 (16.7) | 2 (11.1) |
| Lymphopenia | 0 | 1 (16.7) | 1 (16.7) | 2 (11.1) |

AE, adverse event; ALT, alanine aminotransferase.
