# Supplementary material for: AZD8701, an Antisense Oligonucleotide Targeting FOXP3 mRNA, as Monotherapy and in Combination with Durvalumab: A Phase I Trial in Patients with Advanced Solid Tumors
Source: Clin Cancer Res. 2025 Feb 12;31(8):1449–62. doi: 10.1158/1078-0432.CCR-24-1818 (PMC11995004; doi:10.1158/1078-0432.CCR-24-1818)
Supplement: Supplementary Table S7 — Summary of durvalumab-related adverse events observed in patients treated with AZD8701 + durvalumab [file ccr-24-1818_supplementary_table_s7_suppts7.docx]

## Supplementary materials

**Supplementary Table S7.** Summary of durvalumab-related AEs occurring in >10% of patients with AZD8701 and durvalumab combination therapy.

| **Durvalumab-related AE, n (%)** | **240 mg**  **(*n =* 6)** | **480 mg**  **(*n =* 6)** | **720 mg**  **(*n =* 6)** | **Total**  **(*n =* 18)** |
| --- | --- | --- | --- | --- |
| ALT increased | 0 | 2 (33.3) | 1 (16.7) | 3 (16.7) |
| AST increased | 0 | 2 (33.3) | 1 (16.7) | 3 (16.7) |
| Constipation | 1 (16.7) | 1 (16.7) | 1 (16.7) | 3 (16.7) |
| Diarrhea | 0 | 0 | 2 (33.3) | 2 (11.1) |
| Nausea | 1 (16.7) | 1 (16.7) | 0 | 2 (11.1) |
| Vomiting | 0 | 1 (16.7) | 1 (16.7) | 2 (11.1) |
| Decreased appetite | 0 | 1 (16.7) | 1 (16.7) | 2 (11.1) |
| Lymphopenia | 0 | 1 (16.7) | 1 (16.7) | 2 (11.1) |
| Pruritus | 0 | 0 | 2 (33.3) | 2 (11.1) |
| Pyrexia | 1 (16.7) | 0 | 1 (16.7) | 2 (11.1) |
| Maculo-papular rash | 0 | 0 | 2 (33.3) | 2 (11.1) |

AE, adverse event; ALT, alanine aminotransferase; AST, aspartate aminotransferase.
