# Supplementary material for: AZD8701, an Antisense Oligonucleotide Targeting FOXP3 mRNA, as Monotherapy and in Combination with Durvalumab: A Phase I Trial in Patients with Advanced Solid Tumors
Source: Clin Cancer Res. 2025 Feb 12;31(8):1449–62. doi: 10.1158/1078-0432.CCR-24-1818 (PMC11995004; doi:10.1158/1078-0432.CCR-24-1818)
Supplement: Supplementary Table S8 — Change of liver parameters relative to normal range in patients treated with AZD8701 monotherapy [file ccr-24-1818_supplementary_table_s8_suppts8.docx]

## Supplementary materials

**Supplementary Table S8.** Change of liver parameters relative to normal range: AZD8701 monotherapy.

|  | **60 mg**  **(*n =* 1)** | **120 mg**  **(*n =* 1)** | **240 mg**  **(*n =* 10)** | **480 mg**  **(*n =* 11)** | **720 mg**  **(*n =* 14)** | **960 mg**  **(*n =* 8)** | **Total**  **(*n =* 45)** |
| --- | --- | --- | --- | --- | --- | --- | --- |
| ALP  Shift from normal to low  Shift from normal to high | 0  0 | 0  0 | 0  1 (11.1) | 0  4 (36.4) | 0  5 (35.7) | 0  3 (37.5%) | 0  13 (29.5) |
| ALT  Shift from normal to low  Shift from normal to high | 0  0 | 0  0 | 0  3 (30.0) | 0  4 (36.4) | 0  5 (35.7) | 0  7 (87.5) | 0  19 (42.2) |
| AST  Shift from normal to low  Shift from normal to high | 0  0 | 0  0 | 0  2 (40.0) | 1 (9.1)  5 (45.5) | 0  4 (28.6)) | 0  7 (87.5) | 1 (2.2)  18 (40.0) |
| Bilirubin  Shift from normal to low  Shift from normal to high | 0  0 | 0  0 | 4 (40.0)  0 | 2 (18.2)  1 (9.1) | 2 (14.3)  1 (7.1) | 4 (50.0)  2 (25.0) | 12 (26.7)  4 (8.9) |
| Albumin  Shift from normal to low  Shift from normal to high | 0  0 | 1 (100)  0 | 1 (10.0)  0 | 1 (9.1)  0 | 4 (28.6)  0 | 4 (50.0)  1 (12.5) | 11 (24.4)  1 (2.2) |
| Protein  Shift from normal to low  Shift from normal to high | 0  0 | 0  1 (100) | 3 (30.0)  2 (20.0) | 2 (18.2)  4 (36.4) | 2 (14.3)  2 (14.3) | 3 (37.5)  2 (25.0) | 10 (22.2)  11 (24.4) |
| **Coagulation** | | | | | | | |
| aPPT  Shift from normal to low  Shift from normal to high | 0  0 | 0  0 | 1 (10.0)  3 (30.0) | 4 (36.4)  2 (18.2) | 4 (28.6)  4 (28.6) | 2 (25.0)  3 (37.5) | 11 (24.4)  12 (26.7) |
| Prothrombin intl. normalized ratio  Shift from normal to low  Shift from normal to high | 0  0 | 0  0 | 2 (20.0)  3 (30.0) | 0  0 | 1 (7.1)  4 (28.6) | 1 (12.5)  1 (12.5) | 4 (8.9)  8 (17.8) |
| Prothrombin time  Shift from normal to low  Shift from normal to high | ***n =* 1**  0  0 | ***n =* 1**  0  0 | ***n =* 7**  0  2 (28.6) | ***n =* 6**  0  0 | ***n =* 4**  1 (25.0)  2 (50.0) | ***n =* 3**  0  0 | ***n =* 22**  1 (4.5)  4 (18.2) |

ALP, alkaline phosphatase; ALT, alanine aminotransferase; AST, aspartate aminotransferase; aPPT; activated partial thromboplastin time; intl., international.
