# Supplementary material for: AZD8701, an Antisense Oligonucleotide Targeting FOXP3 mRNA, as Monotherapy and in Combination with Durvalumab: A Phase I Trial in Patients with Advanced Solid Tumors
Source: Clin Cancer Res. 2025 Feb 12;31(8):1449–62. doi: 10.1158/1078-0432.CCR-24-1818 (PMC11995004; doi:10.1158/1078-0432.CCR-24-1818)
Supplement: Supplementary Table S9 — Change of liver parameters relative to normal range in patients treated with AZD8701 + durvalumab [file ccr-24-1818_supplementary_table_s9_suppts9.docx]

## Supplementary materials

**Supplementary Table S9.** Change of liver parameters relative to normal range: AZD8701 and durvalumab combination therapy.

| **Combination therapy** | **240 mg**  **(*n =* 6)** | **480 mg**  **(*n =* 6)** | **720 mg**  **(*n =* 6)** | **Total**  **(*n =* 18)** |
| --- | --- | --- | --- | --- |
| ALP  Shift from normal to low  Shift from normal to high | 0  2 (33.3) | 0  4 (66.7) | 0  5 (83.3) | 0  11 (61.1) |
| ALT  Shift from normal to low  Shift from normal to high | 0  1 (16.7) | 0  4 (66.7) | 0  4 (66.7) | 0  9 (50.0) |
| AST  Shift from normal to low  Shift from normal to high | 0  1 (16.7) | 0  2 (33.3) | 0  4 (66.7) | 0  7 (38.9) |
| Bilirubin  Shift from normal to low  Shift from normal to high | 0  1 (16.7) | 2 (33.3)  1 (16.7) | 1 (16.7)  0 | 3 (16.7)  2 (11.1) |
| Albumin  Shift from normal to low  Shift from normal to high | 1 (16.7)  1 (16.7) | 3 (50.0)  1 (16.7) | 1 (16.7)  1 (16.7) | 5 (27.8)  3 (16.7) |
| Protein  Shift from normal to low  Shift from normal to high | 2 (33.3)  1 (16.7) | 1 (16.7)  1 (16.7) | 2 (33.3)  0 | 5 (27.8)  2 (11.1%) |
| **Coagulation** |  |  |  |  |
| aPPT  Shift from normal to low  Shift from normal to high | 2 (33.3)  0 | 0  1 (16.7) | 3 (50.0)  3 (50.0) | 5 (27.8)  4 (22.2) |
| Prothrombin intl. normalized ratio  Shift from normal to low  Shift from normal to high | 0  1 (16.7) | 0  3 (50.0) | 0  1 (16.7) | 0  5 (27.8) |
| Prothrombin time  Shift from normal to low  Shift from normal to high | 0  0 | 0  2 (66.7) | 0  0 | 1 (14.3)  2 (28.6) |

ALP, alkaline phosphatase; ALT, alanine aminotransferase; AST, aspartate aminotransferase; aPPT; activated partial thromboplastin time; intl., international.
