# Supplementary material for: AZD8701, an Antisense Oligonucleotide Targeting FOXP3 mRNA, as Monotherapy and in Combination with Durvalumab: A Phase I Trial in Patients with Advanced Solid Tumors
Source: Clin Cancer Res. 2025 Feb 12;31(8):1449–62. doi: 10.1158/1078-0432.CCR-24-1818 (PMC11995004; doi:10.1158/1078-0432.CCR-24-1818)
Supplement: Supplementary Table S10 — Detailed response data [file ccr-24-1818_supplementary_table_s10_suppts10.docx]

## Supplementary materials

Supplementary Table S10. Response.

|  | **Monotherapy** | | | | | | | **Combination therapy** | | | |
| --- | --- | --- | --- | --- | --- | --- | --- | --- | --- | --- | --- |
| **Response** | **60 mg (*n =* 1)** | **120 mg (*n =* 1)** | **240 mg (*n =* 10)** | **480 mg (*n =* 11)** | **720 mg (*n =* 14)** | **960 mg (*n =* 8)** | **All  (*N =* 45)** | **240 mg (*n =* 6)** | **480 mg (*n =* 6)** | **720 mg (*n =* 6)** | **All  (*N =* 18)** |
| CR+PR (confirmed and unconfirmed), n (%) 95% CI | 0  0, 97.5 | 0  0, 97.5 | 0  0, 30.8 | 0  0, 28.5 | 0  0, 23.2 | 0  0, 36.9 | 0 0, 7.9 | 0 0, 45.9 | 1 (16.7) 0.4, 64.1 | 1 (16.7) 0.4, 64.1 | 2 (11.1) 1.4, 34.7 |
| CR+PR (objective response), n (%) 95% CI | 0 0, 97.5 | 0 0, 97.5 | 0 0, 30.8 | 0 0, 28.5 | 0  0, 23.2 | 0  0, 36.9 | 0 0, 7.9 | 0 0, 45.9 | 0 0, 45.9 | 1 (16.7) 0.4, 45.9 | 1 (5.6) 0.1 27.3 |
| Median time to response (months) | NA | NA | NA | NA | NA | NA | NA | NA | NA | 5.6 | 5.6 |
| Median duration of response (months) | NA | NA | NA | NA | NA | NA | NA | NA | NA | NA | NA |
| CR+PR+SD (disease control ≥16 weeks) 95% CI | 0  0, 97.5 | 0  0, 97.5 | 4 (40)  12.2, 73.8 | 1 (9.1)  0.2, 41.3 | 6 (42.9)  17.7, 71.1 | 1 (12.5)  0.3, 52.7 | 12 (26.7)  14.6, 41.9 | 1 (16.7)  0.4, 64.1 | 2 (33.3) 4.3, 77.7 | 1 (16.7) 0.4, 64.1 | 4 (22.2) 6.4, 47.6 |
| CR+PR+SD (disease control ≥24 weeks) 95% CI | 0 0, 97.5 | 0 0, 97.5 | 2 (20) 2.5, 55.6 | 1 (9.1) 0.2, 41.3 | 4 (28.6) 8.4, 58.1 | 0 0, 36.9 | 7 (15.6) 6.5, 29.5 | 1 (16.7) 0.4, 64.1 | 1 (16.7) 0.4, 64.1 | 1 (16.7) 0.4, 64.1 | 3 (16.7) 3.6, 41.4 |

CI, confidence interval; CR, complete response; NA, not applicable; PR, partial response; SD, stable disease.
