# Supplementary material for: AZD8701, an Antisense Oligonucleotide Targeting FOXP3 mRNA, as Monotherapy and in Combination with Durvalumab: A Phase I Trial in Patients with Advanced Solid Tumors
Source: Clin Cancer Res. 2025 Feb 12;31(8):1449–62. doi: 10.1158/1078-0432.CCR-24-1818 (PMC11995004; doi:10.1158/1078-0432.CCR-24-1818)
Supplement: Supplementary Table S11 — Pharmacokinetic data [file ccr-24-1818_supplementary_table_s11_suppts11.docx]

## Supplementary materials

**Supplementary Table S11.** Summary of AZD8701 plasma PK parameters (monotherapy and combination) from cycle 1, day 1 and cycle 2, day 22.

|  | | **Dose (QW) – monotherapy (cycle 1, day 1)** | | | | | | | |
| --- | --- | --- | --- | --- | --- | --- | --- | --- | --- |
| **Parameter (units)** | **Statistic** | **60 mg**  **(*n* = 1)** | **120 mg**  **(*n* = 1)** | **240 mg**  **(*n* = 10)** | | **480 mg**  **(*n* = 11)** | **720 mg  (*n*= 14)** | | **960 mg**  **(*n* = 8)** |
| C_max_ | Geometric mean (CV%) |  |  | 31,300 (28.15) | | 47,570 (30.74) | 74,760 (19.14) | | 93,780 (21.98) |
| (ng/mL) | Min–Max [n] | 4,820 [1] | 15,600 [1] | [10] | | [11] | [14] | | [8] |
| AUC_last_ | Geometric mean (CV%) |  |  | 79,330 (23.25) | | 134,700 (30.38) | 228,600 (23.60) | | 308,300 (20.67) |
| (h*ng/mL) | Min–Max [n] | 8,190 [1] | 28,200 [1] | [10] | | [10] | [14] | | [8] |
| t_1/2_lz | Geometric mean (CV%) |  |  | 5.714 (9.785) | | 6.337 (22.34) | 5.895 (13.56) | | 6.207 (11.99) |
| (h) | Min–Max [n] | 8.50 [1] | 8.22 [1] | [10] | | [10] | [14] | | [8] |
| CL | Geometric mean (CV%) |  |  | 3.007 (23.12) | | 3.533 (30.53) | 3.146 (23.57) | | 3.102 (20.66) |
| (L/h) | Min–Max [n] | 7.27 [1] | 4.24 [1] | [10] | | [10] | [14] | | [8] |
| V_ss_ | Geometric mean (CV%) |  |  | 11.21 (15.81) | | 15.95 (29.43) | 13.93 (21.09) | | 16.72 (23.72) |
| (L) | Min–Max [n] | 17.9 [1] | 12.1 [1] | [10] | | [10] | [14] | | [8] |
|  | | **Dose (QW) – monotherapy (cycle 2, day 22)** | | | | | | | |
| **Parameter (units)** | **Statistic** | **60 mg**  **(*n* = 1)** | **120 mg**  **(*n* = 1)** | **240 mg**  **(*n* = 10)** | | **480 mg**  **(*n* = 11)** | **720 mg  (*n =* 14)** | | **960 mg**  **(*n =* 8)** |
| C_max_ | Geometric mean (CV%) |  |  | 33,840 (26.53) | | 42,770 (28.50) | 71,760 (29.26) | | 94,950 (18.12) |
| (ng/mL) | Min–Max [n] | 4,610 [1] | 14,300 [1] | [5] | | [6] | [8] | | [3] |
| AUC_last_ | Geometric mean (CV%) |  |  | 103,300 (34.39) | | 144,100 (18.61) | 261,000 (32.92) | | 357,100 (2.855) |
| (h*ng/mL) | Min–Max [n] | 9,660 [1] | 37,800 [1] | [5] | | [6] | [8] | | [3] |
| t_1/2_lz | Geometric mean (CV%) |  |  | 37.28 (12.02) | | 5.937 (74.56) | 20.58 (131.6) | | 9.095 (155.8) |
| (h) | Min–Max [n] | 59.7 [1] | 33.8 [1] | [5] | | [6] | [8] | | [3] |
| CL | Geometric mean (CV%) |  |  | 2.330 (34.06) | | 3.261 (19.65) | 2.780 (32.26) | | 2.648 (4.341) |
| (L/h) | Min–Max [n] | 6.36 [1] | 3.19 [1] | [5] | | [6] | [8] | | [3] |
| TCP | Geometric mean (CV%) |  |  | 1.232 (11.31) | | 1.111 (10.26) | 1.224 (12.15) | | 1.263 (6.132) |
|  | Min–Max [n] | 1.14 [1] | 1.33 [1] | [5] | | [6] | [8] | | 3] |
|  | | **Dose (QW) – combination (cycle 1, day 1)** | | | | | | | |
| **Parameter (units)** | **Statistic** | **240 mg**  **(*n* = 6)** | | | **480 mg**  **(*n* = 6)** | | | **720 mg**  **(*n*= 6)** | |
| C_max_ | Geometric mean (CV%) | 28,710 (28.10) | | | 53,890 (22.13) | | | 86,680 (9.476) | |
| (ng/mL) | Min–Max [n] | [6] | | | [6] | | | [5] | |
| AUC_last_ | Geometric mean (CV%) | 61,690 (21.20) | | | 156,700 (21.23) | | | 288,300 (16.55) | |
| (h*ng/mL) | Min–Max [n] | [6] | | | [6] | | | [5] | |
| t_1/2_lz | Geometric mean (CV%) | 6.147 (29.27) | | | 6.413 (11.95) | | | 5.801 (7.700) | |
| (h) | Min–Max [n] | [6] | | | [6] | | | [5] | |
| CL | Geometric mean (CV%) | 3.966 (26.41) | | | 3.040 (21.10) | | | 2.495 (16.50) | |
| (L/h) | Min–Max [n] | [6] | | | [6] | | | [5] | |
| V_ss_ | Geometric mean (CV%) | 12.58 (34.61) | | | 13.40 (15.01) | | | 12.92 (9.085) | |
| (L) | Min–Max [n] | [6] | | | [6] | | | [5] | |
|  | | **Dose – combination (cycle 2, day 22)** | | | | | | | |
| **Parameter (units)** | **Statistic** | **3A/240 mg**  **(*n*= 6)** | | | **3A/480 mg**  **(*n* = 6)** | | | **3A/720 mg**  **(*n* = 6)** | |
| C_max_ | Geometric mean (CV%) |  | | |  | | |  | |
| (ng/mL) | Min–Max [n] | 25,100 [1] | | | 60,500–67,500 [2] | | | 64,700 [1] | |
| AUC_last_ | Geometric mean (CV%) |  | | |  | | |  | |
| (h*ng/mL) | Min–Max [n] | 59,700 [1] | | | 178,000-353,000 [2] | | | 192,000 [1] | |
| t_1/2_lz | Geometric mean (CV%) |  | | |  | | |  | |
| (h) | Min–Max [n] | 33.7 [1] | | | 23.6–25.2 [2] | | | 4.05 [1] | |
| CL | Geometric mean (CV%) |  | | |  | | |  | |
| (L/h) | Min–Max [n] | 4.02 [1] | | | 1.36–2.66 [2] | | | 3.68 [1] | |
| TCP | Geometric mean (CV%) |  | | |  | | |  | |
|  | Min–Max [n] | 0.805 [1] | | | 1.18–1.76 [2] | | | 0.761 [1] | |

AUC, area under the concentration-time-curve from the time of dosing to the time of the last measurable concentration; C_max_, maximum concentration; CL, clearance; CV, geometric coefficient of variance (%) Max, maximum; Min, minimum; QW, once per week; t_1/2_lz, terminal half-life; TCP, temporal change parameter; V_ss_, volume of distribution at steady-state.

If geometric mean was not calculated (n<3), the min–max values are displayed instead.
