# Supplementary material for: AZD8701, an Antisense Oligonucleotide Targeting FOXP3 mRNA, as Monotherapy and in Combination with Durvalumab: A Phase I Trial in Patients with Advanced Solid Tumors
Source: Clin Cancer Res. 2025 Feb 12;31(8):1449–62. doi: 10.1158/1078-0432.CCR-24-1818 (PMC11995004; doi:10.1158/1078-0432.CCR-24-1818)
Supplement: Supplementary Figure S1 — Shows trial design and patient disposition [file ccr-24-1818_supplementary_figure_s1_suppfs1.docx]

## Supplementary materials

**Supplementary Figure S1.** **A**) Study design. **B**) Patients treated in the AZD8701 monotherapy dose escalation and PD arms.

A)


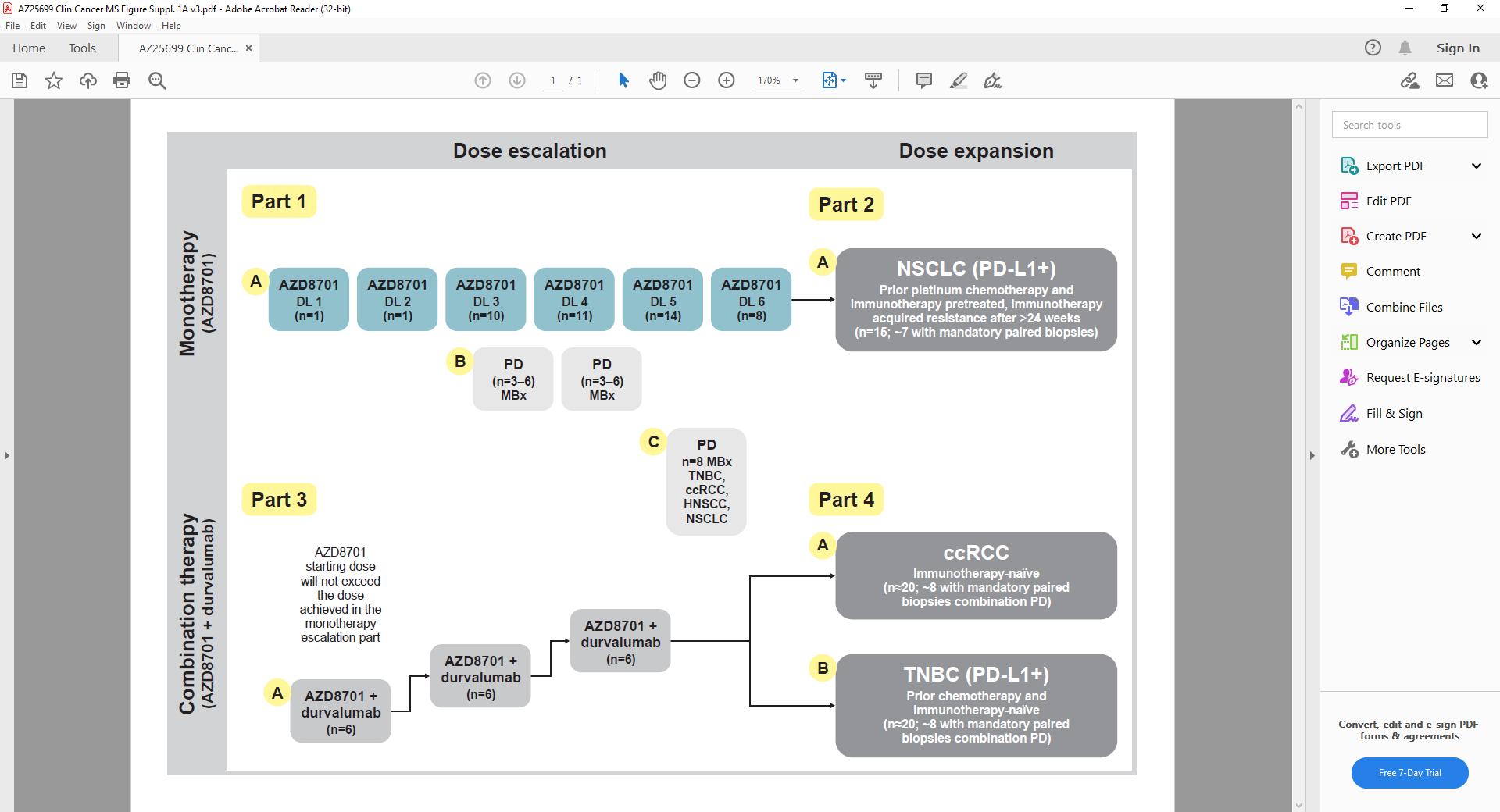


B)

**
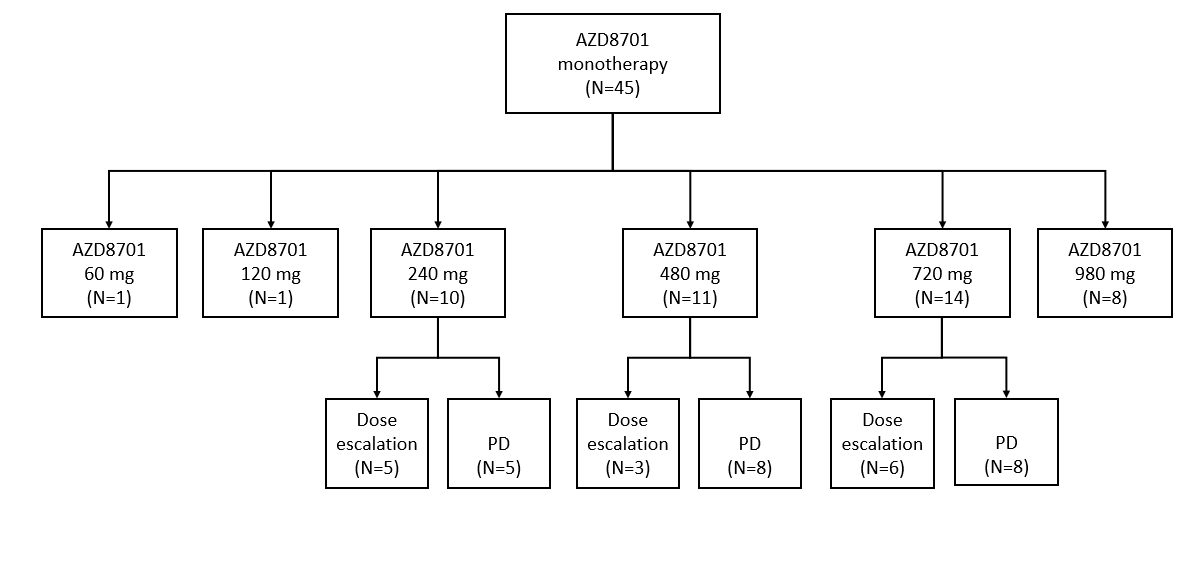
**

ccRCC, clear cell renal cell cancer; HNSCC, head and neck squamous cell carcinoma; MBx, mandatory biopsy; NSCLC, non-small cell lung cancer; PD, pharmacodynamic; PD-L1; programmed cell death protein ligand 1; TNBC, triple-negative breast cancer.

.
