# Supplementary material for: AZD8701, an Antisense Oligonucleotide Targeting FOXP3 mRNA, as Monotherapy and in Combination with Durvalumab: A Phase I Trial in Patients with Advanced Solid Tumors
Source: Clin Cancer Res. 2025 Feb 12;31(8):1449–62. doi: 10.1158/1078-0432.CCR-24-1818 (PMC11995004; doi:10.1158/1078-0432.CCR-24-1818)
Supplement: Supplementary Figure S2 — Shows ASO data and images [file ccr-24-1818_supplementary_figure_s2_suppfs2.docx]

## Supplementary materials

**Supplementary Figure S2.** ISH staining of ASO in pre- and post-treatment biopsy samples. Quantification of ISH staining intensity (normalized to pre-treatment background) in the A) tumor epithelium and B) stroma**.** Note that a patient with epidermoid carcinoma treated with AZD8701 480 mg had no signal in tumor epithelium pre- and post-treatment and a patient with uveal melanoma had no signal in either tumor epithelium or stroma pre- and post-treatment. C) Representative ISH staining images.


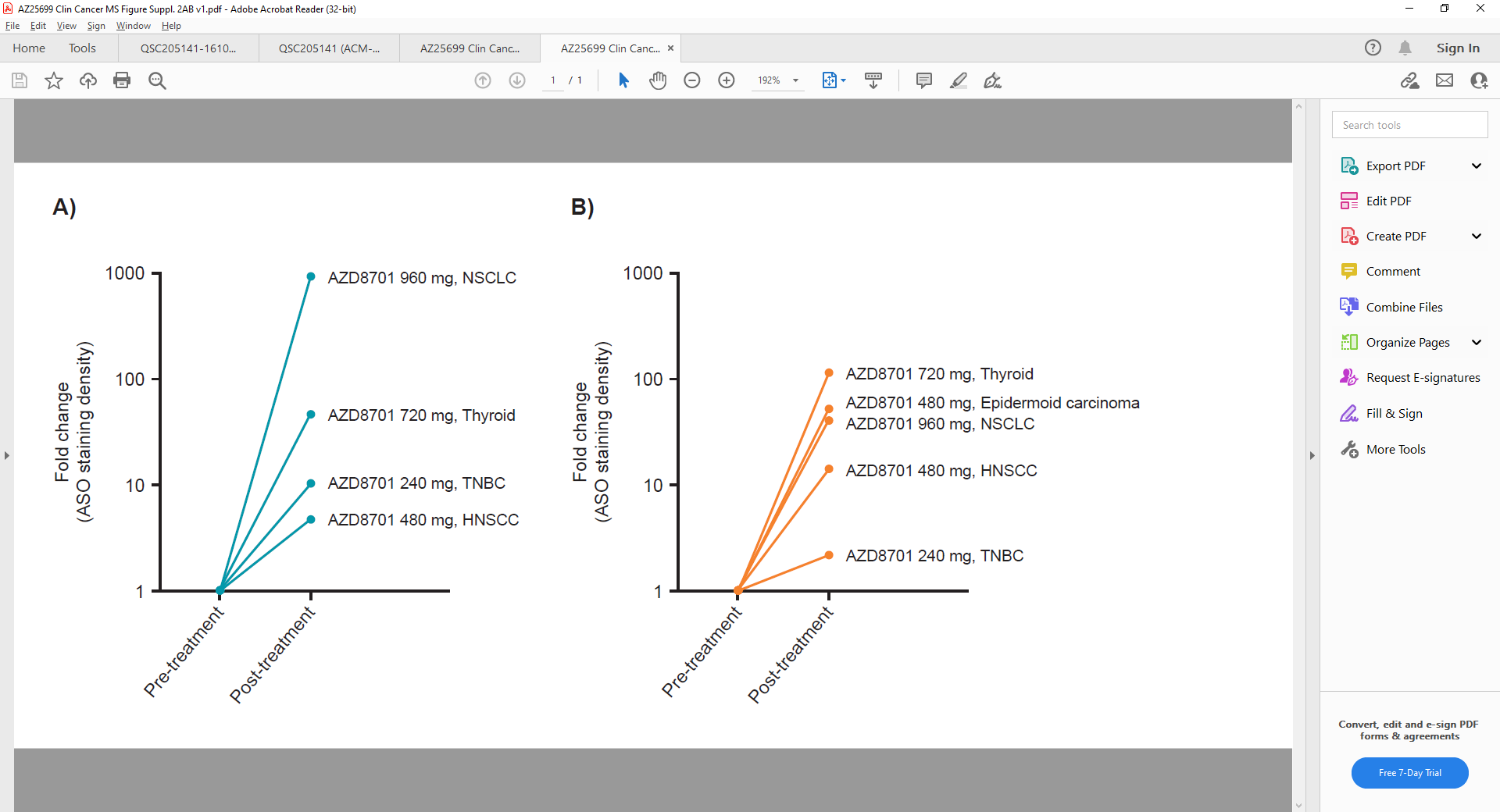


**C)**

**
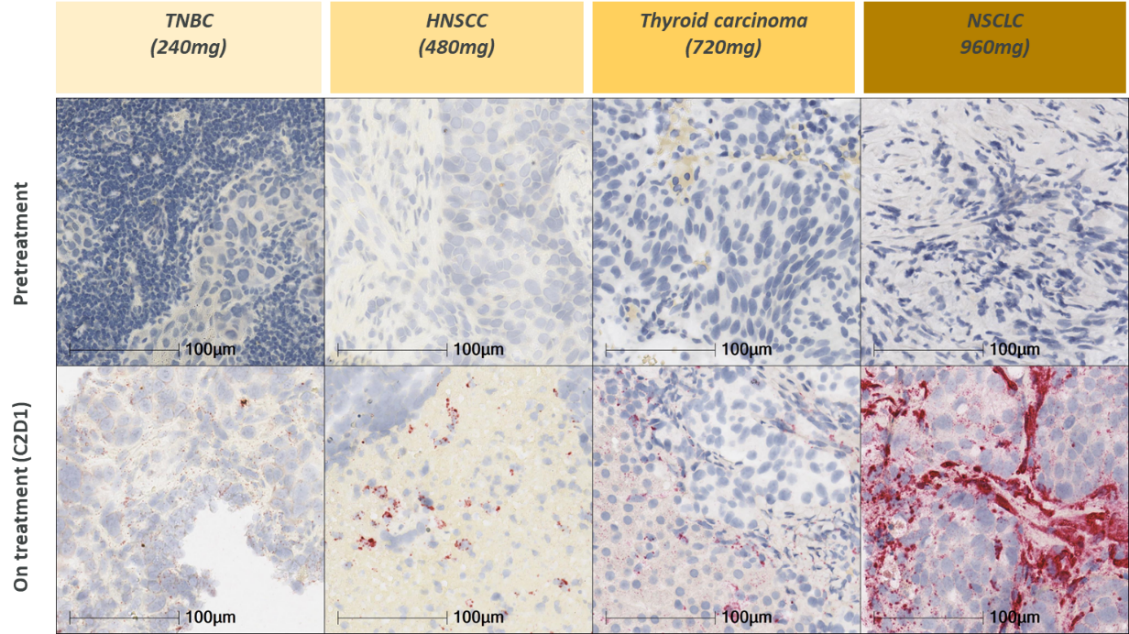
**

ASO, antisense oligonucleotide; HNSCC, head and neck squamous cell carcinoma; ISH, in situ hybridization; NSCLC, non-small cell lung cancer; TNBC, triple-negative breast cancer.
