# Supplementary material for: AZD8701, an Antisense Oligonucleotide Targeting FOXP3 mRNA, as Monotherapy and in Combination with Durvalumab: A Phase I Trial in Patients with Advanced Solid Tumors
Source: Clin Cancer Res. 2025 Feb 12;31(8):1449–62. doi: 10.1158/1078-0432.CCR-24-1818 (PMC11995004; doi:10.1158/1078-0432.CCR-24-1818)
Supplement: Supplementary Material S1 — Definition of dose-limiting toxicities (DLT), Inclusion and exclusion criteria, T, B, and natural killer (TBNK) cell assay, Proliferating T cell assay, miRNAscope in situ hybridization and HALO image analysis [file ccr-24-1818_supplementary_material_s1_suppms1.docx]

## Supplementary materials

### Definition of dose-limiting toxicities (DLTs)

The events below were considered DLTs.

1. ***Hematological events*** ***include***
   1. Neutropenia

- Grade 3 or 4 febrile neutropenia regardless of duration
- Grade 4 neutropenia, without associated fever or systemic infection, lasting for >7 days
- Grade 3 neutropenia, without associated fever or systemic infection, which does not improve by at least one grade within 7 days of onset
  1. Anemia
- Symptomatic grade 3 anemia if associated with clinical sequelae or that requires transfusion of >2 units of red blood cells and assessed as DLT by the Dose Escalation Committee (DEC)
- Grade 4 anemia regardless of duration
  1. Thrombocytopenia
- Grade 3 thrombocytopenia lasting for >7 days
- Grade 3 thrombocytopenia, regardless of duration, associated with bleeding
- Grade 4 thrombocytopenia of any duration
- Grade 3 activated partial thromboplastin time (aPTT) prolongation >72 hours: investigators are requested to repeat coagulation laboratory tests within 72 hours of a Grade 3 aPTT elevation during the DLT period

1. ***Non-hematological events***
   1. Criteria for all patients except patients with elevated transaminases (alanine transaminase [ALT]/aspartate transaminase [AST]) at baseline/screening due to liver metastasis:

- Isolated liver transaminase elevation >5× but ≤10× upper limit of normal (ULN) that does not downgrade to AST/ALT ≤3× ULN or less within 7 days after onset with optimal medical management including systemic corticosteroids
- Transaminase elevation >10× ULN regardless of duration or reversibility
- Isolated total bilirubin (TBL) elevation >3× ULN that does not downgrade to ≤1.5× ULN within 7 days after onset with optimal medical management (for patients with Gilbert’s syndrome: TBL >3 ULN and doubling of direct bilirubin that does not downgrade to baseline value within 7 days)
- Any increase in AST or ALT >3× ULN and concurrent increase in TBL ≥2× ULN, regardless of duration or reversibility, where no reason other than the study intervention can be found to explain the combination of increases (for patients with Gilbert’s syndrome: TBL >3 ULN and doubling of direct bilirubin that does not downgrade to baseline value within 7 days)
  1. DLT criteria only for patients with elevated transaminases (AST/ALT) at baseline/screening due to liver metastasis:
- Isolated liver transaminase elevation >3 × but ≤5 × baseline that does not downgrade to AST/ALT ≤2 × baseline or less within 7 days after onset with optimal medical management including systemic corticosteroids
- Transaminase elevation >5 × baseline regardless of duration or reversibility
- Any increase in AST or ALT >2 × baseline and concurrent increase in TBL ≥2 × ULN (for patients with Gilbert’s syndrome: TBL >3 ULN and doubling of direct bilirubin), regardless of duration or reversibility, where no reason, other than the study intervention, can be found to explain the combination of increases
- Isolated TBL elevation >3 × ULN that does not downgrade to ≤1.5 × ULN within 7 days after onset with optimal medical management (for patients with Gilbert’s syndrome: TBL >3 ULN and doubling of direct bilirubin that does not downgrade to baseline value within 7 days)

1. ***Immune-mediated adverse events*** (imAEs) (see also exclusions)

- Any grade 4 imAE
- Any grade ≥3 non-infectious colitis irrespective of duration after confirmation by colonoscopy and histopathology
- Any grade ≥3 non-infectious pneumonitis irrespective of duration, diagnosis should include clinical evaluation, monitoring of oxygenation using pulse oximetry (resting and exertion), laboratory workup and high-resolution CT scan
- Any grade 3 imAE, including rash, pruritus, or diarrhea (NOTE: this excludes colitis or pneumonitis, as these adverse events [AE]s are already defined above), that does not downgrade to grade ≤2 within 7 days after onset of the event despite maximal supportive care including systemic corticosteroids

1. ***Other events***

- Any death not clearly due to the underlying disease or extraneous causes
- Any other toxicity that is clinically significant and/or unacceptable, and regarded to be a DLT by the DEC

***DLT exclusions***

The following events were DLT exclusions:

- Isolated grade 3 laboratory abnormalities that were not associated with clinical signs or symptoms (e.g., hyperlipasemia or hyperamylasemia not associated with clinical signs or symptoms or radiographic features suggestive of pancreatitis) and are reversed with appropriate maximal medical intervention within 7 days
- Grade 3 or 4 lymphopenia of any duration that is not of clinically significance and that does not require intervention
- Grade 3 inflammatory reaction attributed to a local antitumor response (e.g., inflammatory reaction at sites of metastatic disease, lymph nodes, etc.) that resolves to grade ≤1 within 28 days after onset
- Grade 3 infusion-related reaction: first occurrence and in the absence of steroid prophylaxis that resolves within 12 hours with appropriate clinical management
- Grade 3 rigors, chills, or fever lasting <24 hours with appropriate maximal medical therapy
- Grade 3 diarrhea, nausea, or vomiting that responds to maximal supportive care and improves by at least one grade within 3 days after onset
- Grade 3 fatigue lasting ≤7 days
- Grade 3 endocrine disorder (thyroid, pituitary, and/or adrenal insufficiency) that is managed with or without systemic corticosteroid therapy and/or hormone replacement therapy with resolution of the symptoms within 14 days after treatment onset
- Concurrent vitiligo or alopecia of any AE grade.

### Inclusion and exclusion criteria

#### Inclusion criteria

Patients were eligible to be included in the study only if **all** of the following criteria applied:

- Patient must have been at least 18 years of age at the time of screening
- Bodyweight >35 kg
- Female patients of childbearing potential must have use effective methods of contraception from screening to 15 weeks after the final dose of study intervention
- Non-sterilized male patients who were sexually active with a female partner of childbearing potential had agreed to refrained from fathering a child or donating sperm during the study and for 17 weeks after the final dose of study intervention
- Must have been capable of giving signed informed consent, which included compliance with the requirements and restrictions listed in the ICF and the study protocol
- Eastern Cooperative Oncology Group (ECOG) performance status 0−1 with no deterioration over the previous 2 weeks
- Serum albumin ≥30 g/L
- Life expectancy of ≥12 weeks
- At least one lesion that qualified as a Response Evaluation Criteria in Solid Tumours (RECIST 1.1) target lesion at baseline. Tumor assessment by CT scan or MRI must have been performed within 28 days prior to treatment
- A previously irradiated lesion could have been considered a target lesion if the lesion was well defined, measurable per RECIST 1.1, and had clearly progressed during or after most recent therapy
- Patients undergoing paired tumor biopsies must have additional non-target lesions that can be biopsied at acceptable risk as judged by the investigator or if no other lesion is deemed suitable for biopsy, then a RECIST 1.1 target lesion used for biopsy must be ≥2 cm in longest diameter
- Patients must have been able to provide a formalin-fixed paraffin-embedded specimen or approximately 40, minimum 25, cut sections (prepared less than 12 months prior to enrollment) to be considered for the study
  - If <25 cut sections were available or slides were prepared >12 months prior to enrolment, the Sponsor representative was contacted to discuss.
  - Archival tissue is preferred, but if a participant did not have an archival sample and if a new tissue sample was available (e.g., if it was routine at a site to undertake biopsy of metastatic lesions), the new biopsy specimen could be used
- Adequate organ system functions in the absence of transfusions or growth factor support within 14 days prior to enrollment, as outlined below:
- Absolute neutrophil count ≥1.5×10^9^/L
- Platelets ≥100×10^9^/L
- Hemoglobin ≥9 g/dL
- aPTT ≤1.5× ULN
- Serum bilirubin ≤1.5× ULN. This did not apply to patients with confirmed Gilbert’s syndrome (persistent or recurrent hyperbilirubinemia that was predominantly unconjugated in the absence of hemolysis or hepatic pathology) who were allowed only in consultation with their physician
- AST/ALT ≤2.5× ULN unless liver metastases are present, in which case it must be ≤5× ULN
- Creatinine ≤1.5× ULN, or creatinine clearance ≥45 mL/min as calculated by the Cockcroft–Gault method, or 24-hour measured urine creatinine clearance ≥45 mL/min

Inclusion criteria specific to the dose escalation cohorts:

- Patients with histological or cytological confirmation of a solid, malignant tumor including squamous cell carcinoma of the head and neck (HNSCC), triple-negative breast cancer (TNBC), non-small cell lung cancer (NSCLC), clear cell renal cell carcinoma (ccRCC), gastroesophageal cancer, melanoma, cervical cancer, or small-cell lung cancer (SCLC). Patients with other solid tumors were also eligible if they had had a response to prior anti-PD‑(L)1 treatment (duration of anti‑PD-[L]1 treatment >18 weeks)
- Patients with progressive disease that was refractory to standard therapies or for which no standard therapies existed at the time of the study and a clinical trial was the best option for next treatment based on prior response and/or tolerability to standard of care

Inclusion criteria specific to the monotherapy pharmacodynamic (PD) cohort:

- Patients with histological or cytological confirmation of a solid, malignant tumor including HNSCC, NSCLC, TNBC, or ccRCC
- Patients with progressive disease that was refractory to standard therapies or for which no standard therapies existed at the time of the study and a clinical trial was the best option for next treatment based on prior response and/or tolerability to standard of care

Mandatory paired biopsy patients**:**

- A defined number of patients must have been willing to provide two biopsy samples (pre‑treatment and on-treatment) from a non-target lesion. Patients who provided consent but for whom the fresh biopsy yielded an unsatisfactory tumor sample collection were still eligible
- Patients may have consented to optional biopsies in any dose-escalation cohort prior to cohort expansion for confirmation of PD activity

#### Exclusion criteria

Patients were excluded from the study if **any** of the following criteria applied:

Medical conditions

- Any condition that, in the opinion of the Investigator, would have interfered with evaluation of the study intervention or interpretation of patient safety or study results
- History of allogeneic organ transplantation
- Active or objectively confirmed autoimmune or inflammatory disorders (including inflammatory bowel disease [e.g., ulcerative colitis or Crohn’s disease], diverticulitis [with the exception of diverticulosis], systemic lupus erythematosus, sarcoidosis syndrome, granulomatosis with polyangiitis, Graves’ disease, or rheumatoid arthritis). The following are exceptions to this criterion:
- Patients with vitiligo or alopecia
- Patients with hypothyroidism (e.g. following Hashimoto syndrome) stable on hormone replacement
- Any chronic skin condition that does not require systemic therapy
- Patients without active disease in the last 5 years may be included but only after consultation with the Medical Monitor
- Patients with coeliac disease controlled by diet alone
- Uncontrolled intercurrent illness, including but not limited to, ongoing or active infection, symptomatic congestive heart failure, uncontrolled hypertension, unstable angina pectoris, uncontrolled cardiac arrhythmia, active interstitial lung disease, serious chronic gastrointestinal conditions associated with diarrhea, or psychiatric illness/social situations that would limit compliance with study requirement, substantially increase risk of incurring AEs or compromise the ability of the patient to give written informed consent
- Any of the following cardiac criteria:
- Congestive heart failure per New York Heart Association classification class >II
- Cardiac ventricular arrhythmias requiring anti-arrhythmic therapy.
- Unstable angina or new-onset angina
- Mean QTcF ≥470 ms on screening calculated from three electrocardiograms
- Patients with a history of symptomatic and objectively confirmed arterial (including myocardial infarction) or venous thromboembolic event within 6 months of study entry unless they receive adequate antithrombotic medication
- History of another primary malignancy, except for
- Malignancy treated with curative intent and with no known active disease ≥5 years before the first dose of study intervention and of low potential risk for recurrence
- Adequately treated non-melanoma skin cancer or lentigo maligna without evidence of disease
- Adequately treated carcinoma in situ without evidence of disease
- Active infection including tuberculosis (clinical evaluation that includes clinical history, physical examination and radiographic findings, and tuberculosis testing in line with local practice), hepatitis B (known positive hepatitis B antigen [ HBsAg] result), hepatitis C (HC), or HIV (positive HIV-1/HIV-2 antibodies). Patients with a past or resolved hepatitis B (HB) virus infection (defined as the presence of anti-HBc and absence of HBsAg) are eligible. Patients positive for hepatitis C virus (HCV) antibody are eligible only if polymerase chain reaction is negative for HCV RNA
- Any previous suspected or confirmed COVID-19 diagnosis requiring hospitalization for active respiratory support. In individual cases of previous symptomatic COVID‑19 infection, the eligibility of a patient needs to be discussed with the Medical Monitor
- Current clinical signs and symptoms consistent with COVID-19 (e.g. fever, dry cough, dyspnea, sore throat, fatigue, loss of sense of smell or taste) or confirmed current infection by appropriate laboratory test within the last 4 weeks prior to screening. The clinical signs and symptoms need to be discussed with the Medical Monitor
- Any unresolved toxicity National institute of Health Common Terminology Criteria for Adverse Events (NCI CTCAE) grade ≥1 from previous anticancer therapy with the exception of alopecia, vitiligo, and the laboratory values defined in the inclusion criteria
- Patients with grade ≥2 neuropathy will be evaluated on a case-by-case basis after consultation with the Medical Monitor
- Patients with untreated central nervous system metastases and/or carcinomatous meningitis, identified either on the baseline brain imaging obtained during the screening period or identified prior to signing the informed consent form. Patients whose brain metastases have been treated could participate provided they showed radiographic stability (defined as two brain images, both of which were obtained after treatment to the brain metastases. These imaging scans should both have been obtained at least 4 weeks apart and have not shown evidence of intracranial progression). In addition, any neurologic symptoms that have developed either as a result of the brain metastases or their treatment must have resolved or been stable, either without the use of steroids or on a steroid dose of ≤10 mg/day of prednisone or its equivalent for at least 14 days prior to the start of treatment. Brain metastases were not recorded as RECIST target lesions at baseline
- History of leptomeningeal disease or cord compression
- Known allergy or hypersensitivity to any of the study interventions or any of the study intervention excipients
- History of atypical hemolytic uremic syndrome
- History of active primary immunodeficiency, including a clinical history or prior genetic testing suggesting a mutation in the *FOXP3* gene

Prior/concomitant therapy

- Receipt of the last dose of anticancer therapy (chemotherapy, immunotherapy, endocrine therapy, targeted therapy, biologic therapy, tumor embolization, or monoclonal antibodies [investigational product] within five half-lives or ≤21 days prior to the first dose of study intervention, or palliative radiotherapy within 7 days prior to the first dose of study intervention
- Prior treatment with potential regulatory T cell depletion therapies including agents targeting OX40 or CD357 (GITR) for 90 days prior to enrolment on study
- Patients who have received prior anti-PD-1, anti-PD-L1, or anti- cytotoxic T lymphocyte associated protein 4:
- Must not have experienced a toxicity that led to permanent discontinuation of prior immunotherapy
- All AEs while receiving prior immunotherapy must have had completely resolved or had resolved to baseline prior to screening for this study
- Must not have experienced a grade ≥3 imAE or a neurologic or ocular imAE of any grade while receiving prior immunotherapy. Note: patients with an endocrine AE grade ≤2 were permitted to enroll if they were stably maintained on appropriate replacement therapy and were asymptomatic
- Must not have required the use of additional immunosuppression other than corticosteroids for the management of an AE, not have experienced recurrence of an AE if re-challenged, and not have require current maintenance doses of >10 mg/day prednisone or equivalent
- Current or prior use of immunosuppressive medication within 14 days before the first dose of AZD8701 or durvalumab. The following are exceptions to this criterion:
- Intranasal, inhaled, topical steroids, or local steroid injections (e.g., intra-articular injection)
- Systemic corticosteroids at physiologic doses not exceeding 10 mg/day of prednisone or its equivalent
- Steroids as premedication for hypersensitivity reactions (e.g., CT scan premedication)
- Any concurrent chemotherapy, investigational product, biologic, or hormonal therapy for cancer treatment. Concurrent use of hormonal therapy for non-cancer-related conditions (e.g., hormone replacement therapy) is acceptable
- Radiotherapy treatment to more than 30% of the bone marrow or with a wide field of radiation within 4 weeks of the first dose of study intervention
- Receipt of live attenuated vaccine within 30 days prior to the first dose of study intervention. Note: patients, if enrolled, should not have receive live vaccine while receiving study intervention and up to 30 days after the last dose of the study intervention
- Major surgical procedure (as defined by the Investigator) within 28 days prior to the first dose of study intervention or anticipation of the need for major surgical procedure during the study. Note: Local surgery of isolated lesions for palliative intent was acceptable
- Patients receiving anticoagulation therapy with vitamin K antagonists (e.g., warfarin)

Prior/concurrent clinical study experience

- Had participated in another clinical study with study intervention administered in the last 30 days
- Had concurrent enrolled in another clinical study, unless it was an observational (non‑interventional) clinical study or took place during the follow-up period of an interventional study

Other exclusions

- Female patients who were pregnant or breastfeeding or female patients of reproductive potential who were not willing to employ effective birth control from screening to 15 weeks or 105 days after the last dose of durvalumab or AZD8701 or male patients of reproductive potential for 17 weeks. Female patients should have refrained from breastfeeding and egg donation throughout this period
- Judgement by the investigator that the patient should not participate in the study if the patient is unlikely to comply with study procedures, restrictions, and requirements

### T, B, and natural killer (TBNK) cell assay

For the analysis of T, B, and NK cells, blood collected in tubes containing sodium heparin as an anticoagulant was used. Whole blood (50 μL) was added to a tube containing 18.75 μL of antibody cocktail (CD19 BV421 [BD Bioscience, Cat. No. 562440, RRID AB_11153299], CD4 BV510 [BD Bioscience, Cat. No. 562971, RRID AB_2744424], CD3 FITC [BD Bioscience, Cat. No. 345764, RRID AB_2916364], CD56/CD16 PE [BD Bioscience, Cat. No. 345812/332779, RRID AB_2629216/2868628], CD45 PerCP-Cy5.5 [BD Bioscience, Cat. No. 332784, RRID AB_2868632], CD8 APC [BD Bioscience, Cat. No. 345775, RRID AB_2868803] and CD14 APHC-H7 [BD Bioscience, Cat. No. 641394, RRID AB_1645725]). The tube was briefly vortexed and incubated at room temperature (RT) for 15 minutes protected from light. Subsequently erythrocytes were lysed by the addition of 450 μL of 1x BD FACS™ Lyse solution (BD Biosciences) and incubated at RT protected from light for an additional 15 minutes. Tube contents were then analyzed using a BD FACSCanto™ II flow cytometer (BD Bioscience RRID SCR_018056) running BD FACS™ Diva software (BD Bioscience RRID SCR_001456). Helper and cytotoxic T cell subsets were identified as cells expressing CD4 or CD8, respectively, within the CD3-positive population. Both B cells and NK cells were identified from the non-CD3 expressing population, with the former being positive for CD19 and the latter being positive for CD16 and/or CD56. Monocytes were identified within the leukocyte population by cells expressing CD14. Absolute counts (ABS) were calculated using a dual platform method. The total leukocyte count was calculated by combining the number of events in the lymphocyte gate (SSC vs CD45 PerCP-Cy5.5) with the number of events in the monocyte gate (SSC vs CD14 APC-H7). The lymphocyte:leukocyte ratio was calculated by dividing the number of events in the lymphocyte gate (CD3 BV421 [BD Bioscience, Cat. No. 562426, RRID AB_1152082] vs CD45 PerCP-Cy5.5) by the total leukocyte count. The lymphocyte:leukocyte ratio was multiplied by the white blood cell count from a hematology analyzer. CD14+ monocytes are reported as relative percentages of the total leukocyte count directly from the hematology analyzer.

### Proliferating T cell assay

Whole blood collected in tubes with ACD-B as an anticoagulant was used in the Proliferating T cell subsets assay. 100 μL of whole blood was added to each of two tubes containing a 76 μL monoclonal antibody cocktail comprised of CD3 BV421 (BD Bioscience, Cat. No. 562426, RRID AB_1152082), CD45RA BV510 (BD Bioscience, Cat. No. 563031, RRID AB_2722499), CD197 PE (Biolegend, Cat. No. 353204, RRID AB_10913813), CD45 PerCP-Cy5.5 (BD Bioscience, Cat. No. 332784, RRID AB_2868632), CD8 APC (BD Bioscience, Cat. No. 345775, RRID AB_2868803), and CD4 APC-H7 (BD Bioscience, Cat. No. 6419398, RRID AB_1645732). Tubes were incubated for 20 minutes at room temperature (RT). Subsequently, 1.5 mL of 1x BD FACS™ Lyse solution was added to each tube followed by a 1-second vortex step and a 5-minute incubation period. The vortex and 5-minute incubation were repeated once. Cells were centrifuged at 500 x g for 5 minutes and the supernatant was decanted. Cells were washed by adding 2 mL BD Pharmingen™ Stain Buffer (BSA) and centrifuged at 500 x g for 5 minutes. 500 μL of 1x BD FACS™ Permeabilizing solution 2 was added, the cells were vortexed for 10 seconds and incubated for 10 minutes at RT. Cells were washed by adding 2 mL BD Pharmingen™ Stain Buffer (BSA) and centrifuged at 500 x g for 5 minutes. The supernatant was decanted and 53 μL of antibody cocktail containing BD Horizon™ Brilliant Stain Buffer and IgG1 AF488 (BD Bioscience, Cat. No. 557702, RRID AB_396811) was added to tube 1, and 55 μL of antibody cocktail containing BD Horizon™ Brilliant Stain Buffer and Ki67 AF488 (BD Bioscience, Cat. No. 558616, RRID AB_647087) was added to tube 2. Both tubes were vortexed for 1 second, and cells were incubated for 30 minutes at RT. Cells were washed by adding 2 mL BD Pharmingen™ Stain Buffer (BSA) and centrifuged at 500 x g for 5 minutes, supernatants decanted and resuspended in 300 μL BD Pharmingen™ Stain Buffer (BSA). Cells were analyzed using a BD FACSCanto™ II flow cytometer (RRID SCR_018056) running BD FACSDiva™ software (RRID SCR_001456). Gates were set to identify CD4+ and CD8+ naïve, central memory, effector memory and terminal effector memory subsets. Levels of Ki67 were identified by first applying a gate to each cell population using the IgG1 AF488 isotype control and subsequently applying the same gates to Ki67 AF488 stained cells. Absolute quantities of proliferating T cells were calculated by multiplying the percentages of either CD4+ or CD8+ T cells expressing Ki67 by the CD4+ or CD8+ T cell ABS, respectively, from the TBNK assay.

### miRNAscope in situ hybridization and HALO image analysis

Formalin-fixed, paraffin-embedded sections of pre-treatment and on-treatment (day 1 of cycle 2) biopsies were prebaked to help with tissue adhesion. In situ hybridization (ISH) including dewax, pre-treatment, and hybridization was performed on a Leica Bond RX platform (Leica Biosystems) using the miRNAscope LS Reagent Kit-Red according to the manufacturer’s protocol. A pre-treatment step involved Heat induced epitope retrieval with ER2 and Protease III for 15 minutes each. Probes used for hybridization were positive control SR-RNU6-S1 (Cat. No. 727878-S1) and Target probe SR-ASO-FOXP3-S1 (GATTTTGACATTCTGC). Whole slide images were taken on Aperio AT2 scanner (Leica Biosystems, Cat. No. SS 007056, RRID SCR_021256). HALO image analysis software (HALO v3.4, Indica Labs, Cat. No. PR082, RRID SCR_018350) was used to quantify ISH signal as density (ASO dots/mm^2^) in tumor and stroma regions of the tissue using the ISH module (ISH v4.1.3) and DenseNet (HALO AI) classifier.
